# Normal-Mode-Analysis–Monitored Energy Minimization Procedure for Generating Small–Molecule Bound Conformations

**DOI:** 10.1371/journal.pone.0001025

**Published:** 2007-10-10

**Authors:** Qi Wang, Yuan-Ping Pang

**Affiliations:** Computer–Aided Molecular Design Laboratory, Mayo Clinic, Rochester, Minnesota, United States of America; Center for Genomic Regulation, Spain

## Abstract

The energy minimization of a small molecule alone does not automatically stop at a local minimum of the potential energy surface of the molecule if the minimum is shallow, thus leading to folding of the molecule and consequently hampering the generation of the bound conformation of a guest in the absence of its host. This questions the practicality of virtual screening methods that use conformations at local minima of their potential energy surfaces (local minimum conformations) as potential bound conformations. Here we report a normal-mode-analysis–monitored energy minimization (NEM) procedure that generates local minimum conformations as potential bound conformations. Of 22 selected guest–host complex crystal structures with guest structures possessing up to four rotatable bonds, all complexes were reproduced, with guest mass–weighted root mean square deviations of <1.0 Å, through docking with the NEM–generated guest local minimum conformations. An analysis of the potential energies of these local minimum conformations showed that 22 (100%), 18 (82%), 16 (73%), and 12 (55%) of the 22 guest bound conformations in the crystal structures had conformational strain energies of less than or equal to 3.8, 2.0, 0.6, and 0.0 kcal/mol, respectively. These results suggest that (1) the NEM procedure can generate small–molecule bound conformations, and (2) guests adopt low-strain–energy conformations for complexation, thus supporting the virtual screening methods that use local minimum conformations.

## Introduction

Molecular complexation in biology is best described by the conformational induction theory [1]—namely, a guest binds initially to a less compatible conformation of its host and then adjusts its conformation to induce the most compatible conformation of the host. The conformation induction theory is not ideal for computationally addressing the conformational flexibility of both guest and host in docking studies, however, because computing the mutually dependent conformational changes of both partners on the fly is time–consuming and unsuitable for parallel computing. Alternatively, the conformation selection theory describes that both guest and host select their *preformed* conformations that are most compatible with one another to effect binding by shifting two equilibriums progressively from less compatible to most compatible conformations for both partners [2]–[5]. These preformed and most compatible conformations are conformations at local minima of their potential energy surfaces (local minimum conformations). When the most compatible conformations of both partners are most prevalent, the conformation selection theory becomes the lock–key theory [1]. The conformation selection theory is ideal to computationally account for molecular flexibility in docking because it can convert a guest–host association best described by the conformational induction theory to a series of associations each of which can be described by the lock–key theory [6]. The conformation selection theory thereby affords parallel computing and enables a docking study to be performed using thousands of IBM Blue Gene processors with high processor utilization [6]–[8].

In a recently reported study of 100 small-molecule–protein complex crystal structures, we found that the energy minimization of these small molecules alone does not automatically stop at minima of the potential energy surfaces of these molecules if the minima are shallow, thus leading to the folding of the molecules [9]; we also found that the small–molecule conformations in all 100 crystal structures are nearly identical to their local minimum conformations identified by normal mode analysis [10]–[13] that uses analytic means to analyze harmonic potential wells and classify possible deformations of these molecules according to their energetic costs [9]. These findings suggest that small molecules prefer to adopt local minimum conformations when binding to proteins and theoretically support the virtual screening methods that use local minimum conformations to enable massively parallel docking [6], [8], [14]. In practice, the folding of small molecules caused by energy minimization in the absence of their partners hampers the generation of small–molecule bound conformations from their two–dimensional (2D) structures. This questions the practicality of the virtual screening methods that use local minimum conformations as potential bound conformations.

Herein we report a normal-mode-analysis–monitored energy minimization (NEM) procedure that generates bound conformations of small molecules from their 2D structures and we discuss our test of the NEM procedure. We also report an analysis of conformational strain energies of small–molecule bound conformations. The conformational strain energy is defined herein as the potential energy difference between a conformation of interest and its global minimum conformation. Knowing the strain energy is useful in triaging energetically less stable local minimum conformations in docking studies (see below). Our results suggest that the NEM procedure can generate small–molecule bound conformations and that guests adopt low-strain–energy conformations for complexation, thus offering additional support for the virtual screening methods that use local minimum conformations.

## Results

### Normal-mode-analysis–monitored energy minimization procedure for generating bound conformations

**Figure 1:**
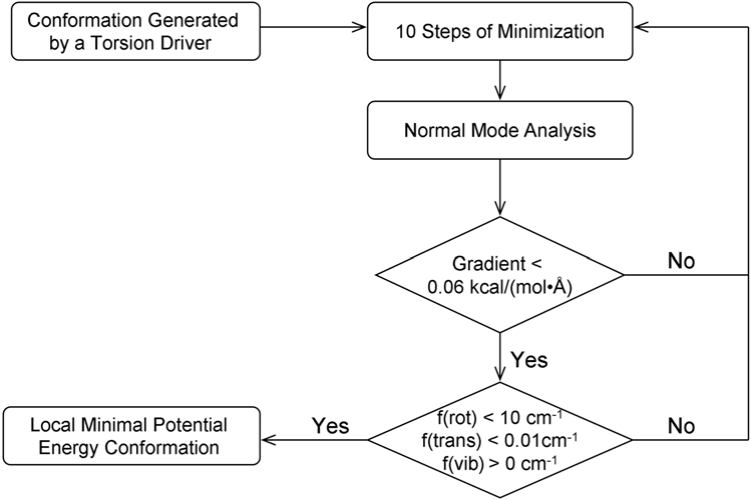
Flowchart of the normal-mode-analysis–monitored energy minimization procedure. mwRMSD stands for mass–weighted root mean square deviation.

As shown in Figure 1, the NEM procedure begins with 10 steps of energy minimization on a guest conformation generated by a torsion driver. The energy–minimized guest conformation is then subject to normal mode analysis to check whether the guest is in its local minimum conformation. The 10–step energy minimization uses a gradient cut-off of 10^−7^ kcal/(mol•Å) and is repeated until the normal mode analysis shows that the guest is in a local minimum conformation. After each 10–step energy minimization, the gradient of the guest potential energy is checked. If the gradient is >0.06 kcal/(mol•Å), the normal mode analysis is aborted, and the guest is considered not to be in its local minimum conformation. If the gradient is ≤0.06 kcal/(mol•Å), the normal mode analysis is performed, and the magnitudes of three translational and three rotational frequencies are checked. If the magnitudes of all translational frequencies are <0.01 cm^−1^ and the magnitudes of all rotational frequencies are <10 cm^−1^, all vibrational frequencies are checked; otherwise, the analysis of vibrational frequencies is aborted and the guest is considered not to be in its local minimum conformation. If all vibrational frequencies are positive, the guest is considered to be in its local minimum conformation [12], [13]. The cut-offs for the gradient and the translational and rotational frequencies are obtained from reference 12, and are based on the fact that geometry cannot be optimized to a gradient of exact zero because of numeric truncations [12]. The NEM procedure is automated by a Perl script shown in Figure S1.

The essence of the NEM procedure is that it generates a local minimum conformation closest to the starting conformation. Different local minimum conformations can therefore be generated from rotamers obtained from the 2D structure by systematically varying the conformation–governing rotatable bonds of the molecule. In theory one of these local minimum conformations is a bound conformation for its particular host molecule according to the conformation selection theory described above.

**Figure 2:**
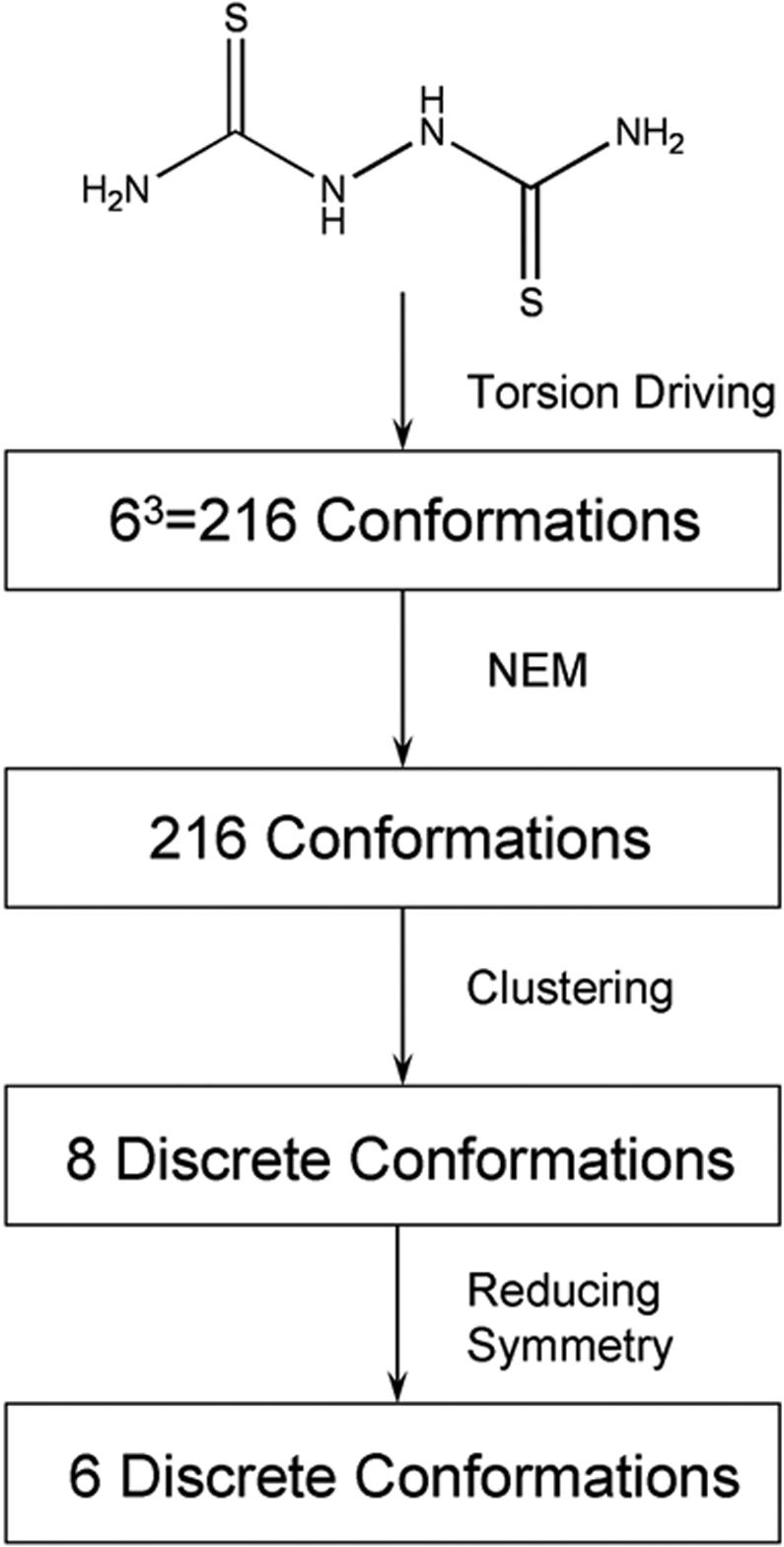
Process for generating the six different local minimum conformations of dithiobiurea used in a docking study to reproduce the dithiobiurea–18-crown-6 crystal structure.

**Figure 3:**
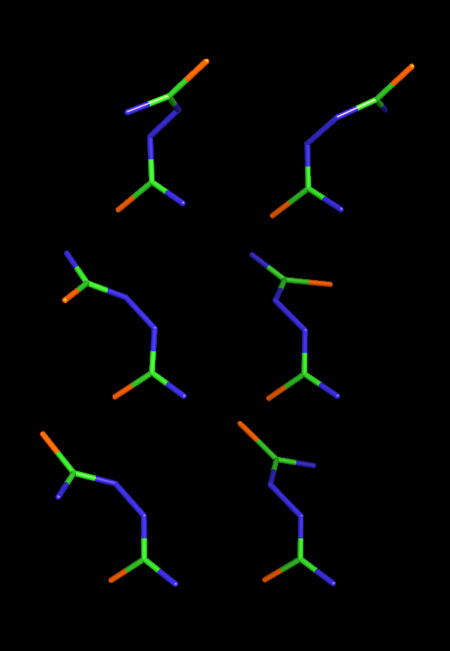
Six different local minimum conformations of dithiobiurea generated by the normal-mode-analysis–monitored energy minimization procedure. The carbon, nitrogen, and sulfur atoms are green, blue, and orange, respectively.

To test whether the NEM procedure can generate a set of guest local minimum conformations one of which is indeed a bound conformation to its known host, the crystal structure of a crown ether 18-crown-6 in complex with dithiobiurea {Cambridge Structural Database (CSD) code [15]: AJUXUY} was used as a model system because dithiobiurea has three conformation–governing rotatable bonds. As shown in Figure 2, 216 rotamers were generated from the 2D structure of dithiobiurea by systematically changing all conformation–governing rotatable bonds of the molecule in increments of 60° of arc starting from 0°. These rotamers were then optimized using the NEM procedure, and a cluster analysis with consideration of molecular symmetry of the 216 optimized rotamers identified six different local minimum conformations of dithiobiurea (Figure 3).

**Figure 4:**
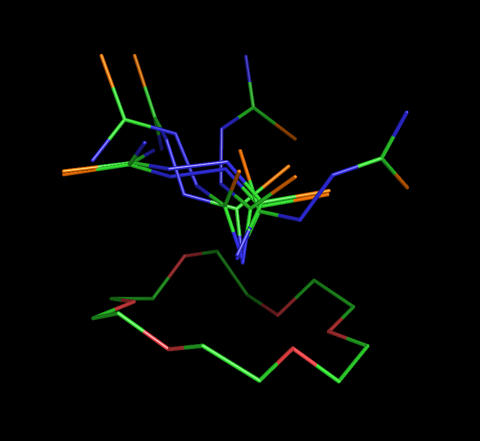
Six energetically indistinguishable dithiobiurea–18-crown-6 complexes generated by the EUDOC program using local minimum conformations of dithiobiurea. The carbon, nitrogen, and sulfur atoms are green, blue, and orange, respectively.

These local minimum conformations were then docked into the three–dimensional (3D) structure of 18-crown-6, which was taken from the complex crystal structure using the EUDOC program [6], [8], [14]. Initially, EUDOC failed to identify the bound conformation of dithiobiurea found in the crystal structure because the differences in the EUDOC–calculated interaction energy among the six dithiobiurea conformations were <0.7 kcal/mol, which is the estimated uncertainty in calculating the interaction energy using EUDOC [6]. Visual inspection of the six EUDOC–generated 18-crown-6–dithiobiurea complexes revealed that there was only one hydrogen bond interaction between 18-crown-6 and dithiobiurea (Figure 4).

**Figure 5:**
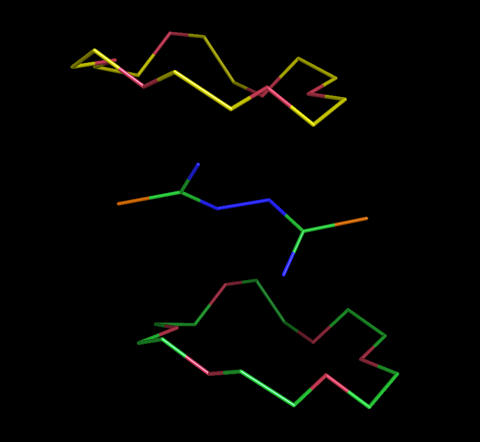
The dithiobiurea–18-crown-6 complex with the strongest intermolecular interaction energy that was identified by the EUDOC program using local minimum conformations of dithiobiurea. The nitrogen and sulfur atoms are blue and orange, respectively. The carbon atoms of the primary and neighboring hosts are green and yellow, respectively.

Repeating the docking study with consideration to the influence of crystal packing on molecular complexation [14], however, identified only one local minimum conformation of dithiobiurea as the bound conformation (Figure 5). The intermolecular interaction energy of this conformation is at least 7.5 kcal/mol lower (stronger) than those of the other five conformations (Table 1); the 18-crown-6 complex with this conformation has a guest mass–weighted root mean square deviation (mwRMSD) of 0.34 Å relative to that of crystal structure AJUXUY. In the 0.34–Å complex, dithiobiurea has both van der Waals and electrostatic interactions with 18-crown-6 as shown by the decomposed interaction energies in Table 1. These results suggested that the NEM procedure could generate a set of guest local minimum conformations one of which is a bound conformation to its host.

**Table 1:** Energies and structural differences of dithiobiurea–18-crown-6 complexes identified by the EUDOC program using six local minimum conformations of dithiobiurea.

Conformation ID^1^	Potential energy (kcal/mol)	Conformational strain energy (kcal/mol)	Interaction energy E_total_ 2 (E_vdw_ 3/E_ele_ 4) (kcal/mol)	mwRMSD^5 ^(Å)
1	0.9	0.0	−24.4 (6.7/−31.1)	1.60
2	0.9	0.0	−13.7 (−7.1/−6.7)	5.69
3	2.9	1.9	−30.2 (−16.3/−13.9)	1.50
4	2.9	1.9	−26.1 (−14.6/−11.9)	1.64
5	4.7	3.8	−41.1 (−19.6/−21.4)	0.34
6	4.7	3.8	−33.6 (−17.0/−16.5)	1.49

1 The IDs of dithiobiurea local minimum conformations generated by the normal-mode-analysis–monitored energy minimization.

2 Intermolecular interaction energy calculated by the EUDOC program.

3 van der Waals component of the intermolecular interaction energy.

4 Electrostatic component of the intermolecular interaction energy.

5 Mass–weighted root mean square deviation of dithiobiurea relative to that of complex crystal structure AJUXUY.

### Testing the normal-mode-analysis–monitored energy minimization procedure

To test the generality of the NEM procedure for generating bound conformations, the validation study with crystal structure AJUXUY was repeated with 21 additional small–molecule complex crystal structures (Table 2). These crystal structures were selected from a previously reported study [14] and have guest structures possessing fewer than five conformation–governing rotatable bonds. The use of this selection criterion reduced the demand for computing resources and allowed better estimation of conformational strain energies of bound conformations (see below). Table 2 lists the results of the validation studies with the 21 crystal structures. The influence of crystal packing was taken into account in all of these studies.

**Table 2:** Accurate reproduction of 22 guest–host complex crystal structures using guest local minimum conformations generated by the normal-mode-analysis–monitored energy minimization procedure.

CSD code^1^	Torsions^2^	E_total_ 3 (kcal/mol)	E_vdw_ 4 (kcal/mol)	E_ele_ 5 (kcal/mol)	Conformations^6^	mwRMSD^7 ^(Å)	E_strain_ 8 (kcal/mol)
AJUXOS	1	–31.7	–15.3	−16.4	2	0.39	0.0
AJUXUY	3	−41.1	−19.6	−21.4	6	0.34	3.8
AJUYAF	3	−35.4	−18.4	−17.0	6	0.52	3.8
BAFZEN	1	−202.1	4.2	−206.2	3	0.12	0.0
BAPRAM	4	−42.0	−17.5	−24.5	24	0.64	2.0
BAPREQ	4	−37.0	−24.1	−12.9	20	0.30	2.0
BEGVOZ	2	−68.4	−16.9	−51.5	5	0.30	2.7
CECMEC10	3	−36.5	−23.6	−12.9	11	0.26	0.0
DESHEO	1	−50.5	−12.1	−38.4	2	0.25	0.0
DOXWAO	3	−76.2	−29.0	−47.2	8	0.36	0.0
FANJAG	3	−35.3	−24.6	−10.7	12	0.18	0.1
GUGGUK	1	−185.7	−6.3	−179.4	3	0.30	0.5
HASWUT	2	−229.3	−17.1	−212.2	4	0.22	0.0
JEJWOK	2	−29.4	−25.2	−4.2	3	0.58	0.0
KAXPOO	4	−39.6	−29.5	−10.1	26	0.52	0.0
LAYMAZ	3	−66.2	−10.5	−55.7	12	0.60	0.6
NOYNAQ	3	−28.7	−12.8	−15.9	18	0.41	3.2
OCAMIO	2	−23.8	−17.4	−6.4	6	0.62	0.0
UBETAW	4	−62.8	−30.1	−32.7	6	0.46	0.0
VOHVIX	3	−47.9	−27.8	−20.2	19	0.38	0.0
XIVVAZ	3	−106.1	−8.7	−97.4	63	0.70	0.4
YACVEE	2	−29.8	−19.7	−10.1	6	0.35	0.0

1 Cambridge Structural Database codes of the 22 slected guest-host complex crystal structures.

2 Number of conformation-governing torsions of the guest.

3 Intermolecular interaction energy calculated by the EUDOC program.

4 van der Waals component of the intermolecular interaction energy.

5 Electrostatic component of the intermolecular interaction energy.

6 Number of different guest local minimum conformations obtained using the normal-mode-analysis–monitored energy minimization (NEM) procedure.

7 Mass–weighted root mean square deviation of the host-bound guest obtained by using the NEM procedure relative to the corresponding crystal structure.

8 Conformational strain energy of the host–bound guest conformation.

Of the 22 small–molecule complex crystal structures, including complex AJUXUY described above, 22 and 15 of them were produced with guest mwRMSDs of less than 1.0 and 0.5 Å, respectively by docking the NEM–generated guest local minimum conformations into their host structures that were taken from the corresponding complex crystal structures (Table 2). These results show that the NEM procedure can generate bound conformations in the absence of their host structures regardless of the number of local minimum conformations or the molecular complexity. It also demonstrates the generality of the NEM procedure for generating small–molecule bound conformations.

### Analysis of conformational strain energies of bound conformations

In this study, the number of conformation–governing rotatable bonds in all 22 guest structures was fewer than five, and the rotamers were optimized using the NEM procedure. Sampling of guest conformations and identification of the global minimum conformation could therefore be done at a relatively fine granularity. Accordingly, the conformational strain energies of the 22 bound guest conformations in the complex crystal structures were determined from the potential energy difference between the EUDOC–identified bound conformation and the global minimum conformation. Of the 22 guest bound conformations in the guest-host complex crystal structures studied, 22 (100%), 18 (82%), and 16 (73%) of them have the conformational strain energies of less than or equal to 3.8, 2.0, and 0.6 kcal/mol, respectively (Table 2); 12 of them (55%) are in their global minimum conformations.

## Discussion

### The conformation sampling resolution

In this study, a cluster width of 60° of arc was used in cluster analysis. Although this width has been widely used and proven adequate for sampling the energy landscape of small to medium size molecules [16], [17], it was desirable to confirm that this cluster width is narrow enough to identify distinct local minimum conformations. In repeating the cluster analysis and subsequent docking studies for the 22 complexes (see above) using a cluster width of 30° of arc, we identified new guest local minimum conformations for only two complexes (CSD codes: CECMEC10 and DOXWAO), but found that none of the new conformations have a lower potential energy than the global minimum conformation that was identified with the cluster width of 60° of arc and that none of these new conformations can form a complex with an interaction energy that is stronger than that of the complex obtained with the cluster width of 60° of arc. These results confirm that the 60°–of–arc conformation sampling resolution is adequate for generating distinct local minimum conformations.

### The conformational strain energies of the bound conformations

The analysis of the conformational strain energies described above showed that 22 (100%), 18 (82%), 16 (73%), and 12 (55%) of the 22 guest bound conformations in the guest-host complex crystal structures have the conformational strain energies of less than or equal to 3.8, 2.0, 0.6, and 0.0 kcal/mol, respectively (Table 2). These observations are consistent with the report that approximately 70% of the small–molecule bound conformations in their protein-bound crystal structures have conformational strain energies of ≤3.0 kcal/mol [18]. These data are also consistent with our recently reported study of six small–molecule–protein complex crystal structures [9] in which 6 (100%), 5 (83%), 4 (67%), and 1 (17%) small–molecule bound conformations have the conformational strain energies of less than or equal to 2.3, 1.5, 0.88, and 0 kcal/mol, respectively. In this context, we propose to use a cut-off of 5.0 kcal/mol for the conformational strain energy to triage energetically less stable local minimum conformations in docking studies. This cut-off can significantly reduce the number of conformations used in a docking study and shorten the computing time for docking. For example, for the guest structure in one of the 22 complexes (CSD code: BAPRAM), rotamer generation, optimization with the NEM procedure, and conformational clustering identified 24 different local minimum conformations, but only eight of them (33%) need to be docked if a conformational strain energy cut-off of 5.0 kcal/mol is used to remove energetically less stable local minimum conformations. It is conceivable that using this cut-off the number of local minimum conformations will be markedly reduced, thus shortening the docking process greatly, when molecules to be docked have more than five conformation–governing rotatable bonds. Although the cut-off of 5.0 kcal/mol is a good starting point, more studies of various molecular complexes are needed to refine it.

### Generality of the NEM procedure for generating bound conformations

In this study the NEM procedure was used in conjunction with a rotamer sampling approach to generate local minimum conformations of a molecule possessing fewer than five conformation–governing rotatable bonds. Given the U.S. National Science Foundation's petascale science and engineering initiative (http://www.nsf.gov/pubs/2005/nsf05625/nsf05625.htm) and the current cost reduction rate for disk space, it is conceivable that generation of large numbers of local minimum conformations for a molecule with more than four conformation–governing rotatable bonds is computationally feasible, because the calculations to search for different local minimum conformations are embarrassingly parallel over the commodity–driven multicore/multithread computer hardware. The NEM procedure can be applied to other conformation sampling approaches as well. When the number of conformation–governing rotatable bonds of a molecule is too large (e.g., >10), the rotamer sampling approach can be computationally expensive. In that case, other approaches such as distance/conformational constraints [16], radial or adaptive sampling technique [19], or stochastic sampling with multiple molecular dynamics simulations [20]–[25] can be used in conjunction with the NEM procedure to generate local minimum conformations. Given our finding that small molecules prefer to adopt local minimum conformations when binding to their partners [9], the NEM procedure, which can generate a local minimum conformation closest to the starting conformation, appears to be a plausible procedure for generating small–molecule bound conformations that are useful for docking studies and for large–scale virtual screening of chemical databases for drug leads [7], [8].

## Methods

### Selection of the 22 guest–host complex crystal structures

We selected 22 guest–host complex crystal structures from a published study of 161 small–molecule complex crystal structures, all of which were reproduced with guest mwRMSDs of <1.0 Å by the EUDOC program using the bound conformations of guests and hosts taken from crystal structures [14]. The selection criterion was that the number of conformation–governing rotatable bonds was fewer than five. The conformation–governing rotatable bond is defined as a torsion whose rotation changes the conformation of the molecule. A terminal torsion (e.g., the torsion of CH_3_CH_3_) is generally considered not to be a conformation–governing rotatable bond; however, a terminal torsion comprising the OH group or the F atom is treated as a conformation–governing rotatable bond because the hydroxyl H or F atom is a hydrogen bond donor or acceptor, respectively. The CSD codes for the 22 selected complexes are listed in Table 2.

### Preparation of the bound conformations of the hosts

The 22 guest–bound host conformations were taken from the guest–host complex crystal structures. The atomic charges of these hosts were generated according to the RESP procedure [26] with *ab initio* calculations at the HF/6-31G* level using the Gaussian 98 program [27] (Table S1). The force field parameters of these hosts were generated using the ANTECHAMBER module of the AMBER 7 program [28] using the Cornell et al. force field (parm99.dat/gaff.dat) [29] (Table S2).

### Generation of the local minimum conformations of the guests

For each guest structure, a set of local minimum conformations was generated according to the following steps: (1) A 2D structure was converted to a 3D structure using the QUANTA97 program (Accelrys Software, Inc, San Diego, California). The atomic charges of the 3D structure were generated using the same method used for generating the host charges. (2) New conformations of the 3D structure were generated by systematically changing all conformation–governing rotatable bonds using the INTERFACE module of the AMBER 5 program [28] at a torsion increment of 60° of arc starting from 0°. The INTERFACE module generated 6^n^ conformations in total, where n is the number of conformation–governing rotatable bonds. The torsional restraints used by the module were set as parabolic to the designated angle ±40° of arc and linear sides beyond that torsion range. The force constant used to restrain the conformation–governing rotatable bonds was 50 kcal/(mol•rad^2^). (3) Each conformation generated by the INTERFACE module was then subjected to the NEM procedure for energy minimization. (4) Cluster analysis was performed on each conformation–governing rotatable bond of the energy–minimized conformations. Each cluster contains all the conformations each of which has a torsion angle within ±30° of the average values of all the members in the corresponding cluster (cluster center). (5) One conformation was randomly chosen from each cluster as a representative conformation.

### Docking studies using the EUDOC program

The algorithm of the EUDOC program has been reported elsewhere [6]. Briefly, it uses a systematic search protocol, translating and rotating a guest in a putative binding pocket of a host and repeating the translations and rotations with different conformations of both guest and host to search for energetically favorable conformations, orientations, and positions of the guest relative to the host. A docking box is defined within the binding pocket to confine the translation of the guest. The intermolecular interaction energy is the potential energy of the guest–host complex relative to the potential energies of the two partners in their free states. This energy is calculated according to Equations 1 and 2 using the second–generation AMBER force field [29]. In calculating the intermolecular interaction energy, the multiplicative dielectric constant is set to 1.0, and the distance cut-offs for steric and electrostatic interactions are set to 10^9^ Å.
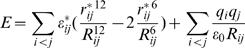
(Eq.1)

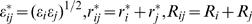
(Eq.2)In this study, a docking box was defined to enclose the guest structure in the host structure; each dimension of the box is ≥6 Å, the size of the docking box and the cut-off for the interaction energy used by the EUDOC program are listed in Table S3; the complex–prediction module of EUDOC was used to translate and rotate the guest around the host at increments of 1.0 Å and 10° of arc, respectively; all different guest local minimum conformations were automatically docked into the host structure taken from the corresponding guest–host complex crystal structure using EUDOC.

To consider the influence of crystal packing, the PyMOL program (DeLano Scientific LLC, South San Francisco, California) was used to generate a multimeric host system by applying the symmetry of the space group of the crystal structure. Host or guest structures were excluded from the multimeric host system if the shortest distance of a heavy atom of the guest structure to be docked to the heavy atom of the host/guest structure in neighboring unit cells was >4.0 Å.

### Energy minimization monitored with normal mode analysis

Energy minimization used (1) 10^6 ^steps of energy minimization, (2) a dielectric multiplicative constant of 80.0, (3) the steepest descent or conjugate gradient method, (4) a nonbonded cut-off of 12 Å, (9) a 10^−7^-kcal/(mole•Å) cut-off for the root-mean-square of the Cartesian elements of the gradient, and (10) defaults for other inputs of the SANDER module of the AMBER 5 program [28]. NMA used (1) a dielectric multiplicative constant of 80.0, (2) a nonbonded cut-off of 12 Å, and (3) defaults for other inputs of the NMODE module of the AMBER 8 program [28].

### Mass–weighted root mean square deviations

The mwRMSDs were calculated by superimposing the host portion of the EUDOC–generated complex over the corresponding host portion of the crystal structure. The mwRMSD of all atoms of the guest portion in the two superimposed complexes were determined using the PTRAJ module of the AMBER 8 program [28].

## Supporting Information

Figure S1 Perl script for the normal-mode-analysis-monitored energy minimization procedure.
(0.05 MB PDF)

Table S1 The RESP charges and the AMBER atom types of 22 host-guest complexes. Atom names, the AMBER atom types, Cartesian coordinates x,y and z, and the RESP charges are at columns 3, 4, 6, 7, 8 and 9. Suffixes “h” and “g” specifies the host and the guest of the complex crystal structure.
(0.34 MB PDF)

Table S2 The AMBER force field parameters for the 22 guest structures.
(0.02 MB PDF)

Table S3 Detailed information with regard to the docking studies of the 22 host-guest complexes.
(0.02 MB PDF)
