# Supplementary material for: Normal-Mode-Analysis–Monitored Energy Minimization Procedure for Generating Small–Molecule Bound Conformations
Source: PLoS One. 2007 Oct 10;2(10):e1025. doi: 10.1371/journal.pone.0001025 (PMC1995756; doi:10.1371/journal.pone.0001025)
Supplement: Table S1 — The RESP charges and the AMBER atom types of 22 host-guest complexes. Atom names, the AMBER atom types, Cartesian coordinates x,y and z, and the RESP charges are at columns 3, 4, 6, 7, 8 and 9. Suffixes “h” and “g” specifies the host and the guest of the complex crystal structure. (0.34 MB PDF) [file pone.0001025.s002.pdf]

Table S1. The RESP charges and the AMBER atom types of 22 host-guest complexes. Atom names, the AMBER atom types, Cartesian coordinates x,y and z, and the RESP charges are at columns 3, 4, 6, 7, 8 and 9. Suffixes “h” and “g” specifies the host and the guest of the complex crystal structure.

AJUXOSg

1	0	N1	N	M	6.3750	1.7500	0.1800	-0.5460
2	1	H1	H	E	6.6830	2.3690	0.6400	0.3360
3	1	H2	H	E	5.7790	1.9230	-0.3700	0.3360
4	1	C1	CM	M	6.7090	0.5170	0.4160	0.2610
5	4	S1	SS	E	7.8570	0.0570	1.5440	-0.3870
6	4	C2	CM	M	5.9450	-0.5170	-0.4160	0.2610
7	6	S2	SS	E	4.7970	-0.0570	-1.5440	-0.3870
8	6	N2	N	M	6.2790	-1.7500	-0.1800	-0.5460
9	8	H3	H	E	5.9710	-2.3690	-0.6400	0.3360
10	8	H4	H	E	6.8750	-1.9230	0.3700	0.3360

AJUXUYh

1	0	C1	CT	M	12.4450	7.2400	-5.3850	0.0830
2	1	H1	H1	E	12.6790	8.0740	-5.8240	0.0370
3	1	H2	H1	E	12.3750	6.5480	-6.0610	0.0370
4	1	C2	CT	M	13.4950	6.8760	-4.3860	0.0830
5	4	H3	H1	E	13.2800	6.0270	-3.9700	0.0370
6	4	H4	H1	E	14.3570	6.7940	-4.8240	0.0370
7	4	O2	OS	M	13.5400	7.9080	-3.4030	-0.3150
8	7	C3	CT	M	14.4500	7.6130	-2.3510	0.0830
9	8	H5	H1	E	15.3580	7.5970	-2.6920	0.0370
10	8	H6	H1	E	14.2500	6.7430	-1.9730	0.0370
11	8	C4	CT	M	14.3140	8.6710	-1.3030	0.0830
12	11	H7	H1	E	15.0330	8.5920	-0.6560	0.0370
13	11	H8	H1	E	14.3630	9.5500	-1.7100	0.0370
14	11	O3	OS	M	13.0540	8.5050	-0.6530	-0.3150
15	14	C5	CT	M	12.7300	9.6030	0.1850	0.0830
16	15	H9	H1	E	12.6830	10.4170	-0.3400	0.0370
17	15	H10	H1	E	13.4170	9.7160	0.8600	0.0370
18	15	C6	CT	M	11.4090	9.3430	0.8400	0.0830
19	18	H11	H1	E	11.4580	8.5350	1.3750	0.0370
20	18	H12	H1	E	11.1780	10.0830	1.4220	0.0370
21	18	O4	OS	M	10.4210	9.1950	-0.1750	-0.3150
22	21	C7	CT	M	9.1120	9.0990	0.3820	0.0830
23	22	H13	H1	E	8.8000	9.9790	0.6450	0.0370
24	22	H14	H1	E	9.1300	8.5350	1.1700	0.0370
25	22	C8	CT	M	8.1870	8.5170	-0.6370	0.0830
26	25	H15	H1	E	8.4720	7.6180	-0.8660	0.0370
27	25	H16	H1	E	7.2890	8.4660	-0.2740	0.0370
28	25	O5	OS	M	8.1900	9.3350	-1.8060	-0.3150
29	28	C9	CT	M	7.1740	8.9620	-2.7350	0.0830
30	29	H17	H1	E	7.2010	9.5810	-3.4820	0.0370
31	29	H18	H1	E	6.3110	9.0680	-2.3020	0.0370
32	29	C10	CT	M	7.2630	7.5450	-3.2840	0.0830
33	32	H19	H1	E	7.0820	6.9260	-2.5590	0.0370
34	32	H20	H1	E	6.5610	7.4300	-3.9450	0.0370
35	32	O6	OS	M	8.5070	7.1810	-3.8840	-0.3150
36	35	C11	CT	M	8.9250	8.0890	-4.8970	0.0830
37	36	H21	H1	E	9.1500	8.9440	-4.4960	0.0370
38	36	H22	H1	E	8.2010	8.2320	-5.5250	0.0370
39	36	C12	CT	M	10.1180	7.5430	-5.6190	0.0830
40	39	H23	H1	E	9.8970	6.6880	-6.0200	0.0370
41	39	H24	H1	E	10.3780	8.1510	-6.3280	0.0370
42	39	O1	OS	M	11.1980	7.3810	-4.7010	-0.3150

AJUXUYg

1	0	S1	SS	M	11.0570	3.8660	-5.4410	-0.4160
2	1	C1	CM	M	10.7400	3.7840	-3.7920	0.0080
3	2	N1	N	B	11.3020	2.9130	-2.9600	-0.3480
4	3	H1	H	E	11.8000	2.3360	-3.2760	0.2800
5	3	H2	H	E	10.9890	2.7960	-2.1510	0.2800

6	2	N2	N	M	9.8200	4.6350	-3.2550	-0.0330
7	6	H3	H	E	9.5370	5.2250	-3.6950	0.2290
8	6	N3	N	M	9.6340	4.7560	-1.8840	-0.0330
9	8	H4	H	E	9.2140	4.1610	-1.5430	0.2290
10	8	C2	CM	M	10.3780	5.6150	-1.1330	0.0080
11	10	N4	N	B	11.1620	6.4610	-1.7870	-0.3480
12	11	H5	H	E	11.1090	6.5550	-2.6280	0.2800
13	11	H6	H	E	11.6010	7.0720	-1.3320	0.2800
14	10	S2	SS	M	10.2450	5.5770	0.5470	-0.4160

AJUYAFh

1	0	O1	OS	M	14.7810	7.2560	12.8140	-0.3170
2	1	C1	CT	M	15.5480	6.2180	13.4190	0.1680
3	2	H1	H1	E	16.3520	6.0610	12.9010	0.0180
4	2	H2	H1	E	15.0320	5.3970	13.4350	0.0180
5	2	C3	CT	M	15.9180	6.6170	14.8270	0.0470
6	5	H4	H1	E	16.5240	5.9610	15.2060	0.0410
7	5	H5	H1	E	16.3660	7.4770	14.8190	0.0410
8	5	O2	OS	M	14.7330	6.6950	15.6160	-0.2800
9	8	C8	CT	M	15.0240	6.9720	16.9830	0.0470
10	9	H13	H1	E	15.5880	7.7580	17.0500	0.0410
11	9	H14	H1	E	15.4960	6.2230	17.3790	0.0410
12	9	C11	CT	M	13.7290	7.2060	17.7150	0.1680
13	12	H19	H1	E	13.9010	7.3670	18.6550	0.0180
14	12	H20	H1	E	13.2740	7.9800	17.3490	0.0180
15	12	O4	OS	M	12.9280	6.0470	17.5560	-0.3170
16	15	C13	CT	M	11.6010	6.1770	18.0930	0.0150
17	16	H23	H1	E	11.1890	7.0000	17.7570	0.0770
18	16	C15	CT	M	11.6110	6.1960	19.6250	-0.0660
19	18	H26	HC	E	12.1250	6.9560	19.9380	0.0340
20	18	H27	HC	E	10.7030	6.2890	19.9560	0.0340
21	18	C18	CT	M	12.2230	4.9040	20.1660	0.0320
22	21	H31	HC	E	12.1950	4.9150	21.1350	-0.0010
23	21	H32	HC	E	13.1520	4.8490	19.8930	-0.0010
24	21	C20	CT	M	11.4720	3.6900	19.6500	0.0320
25	24	H35	HC	E	10.5720	3.6940	20.0110	-0.0010
26	24	H36	HC	E	11.9140	2.8840	19.9600	-0.0010
27	24	C19	CT	M	11.4020	3.6700	18.1170	-0.0660
28	27	H33	HC	E	12.2920	3.5480	17.7510	0.0340
29	27	H34	HC	E	10.8510	2.9260	17.8240	0.0340
30	27	C16	CT	M	10.8120	4.9770	17.6080	0.0150
31	30	H28	H1	E	9.9010	5.0540	17.9610	0.0770
32	30	O6	OS	M	10.7370	5.0630	16.1800	-0.3170
33	32	C17	CT	M	9.8080	4.1650	15.5750	0.1680
34	33	H29	H1	E	8.9700	4.1770	16.0640	0.0180
35	33	H30	H1	E	10.1600	3.2620	15.5930	0.0180
36	33	C14	CT	M	9.5800	4.6010	14.1490	0.0470
37	36	H24	H1	E	9.2660	5.5180	14.1310	0.0410
38	36	H25	H1	E	8.9040	4.0400	13.7360	0.0410
39	36	O5	OS	M	10.8040	4.4930	13.4260	-0.2800
40	39	C12	CT	M	10.5950	4.8090	12.0480	0.0470
41	40	H21	H1	E	9.9500	4.1930	11.6660	0.0410
42	40	H22	H1	E	10.2380	5.7080	11.9700	0.0410
43	40	C10	CT	M	11.8970	4.7130	11.2880	0.1680
44	43	H17	H1	E	11.7190	4.6530	10.3370	0.0180
45	43	H18	H1	E	12.3790	3.9160	11.5590	0.0180
46	43	O3	OS	M	12.6790	5.8620	11.5590	-0.3170
47	46	C5	CT	M	13.9690	5.8490	10.9400	0.0150
48	47	H8	H1	E	14.4770	5.0720	11.2560	0.0770
49	47	C2	CT	M	14.6680	7.1340	11.3880	0.0150
50	49	H3	H1	E	15.5700	7.1410	11.0070	0.0770
51	49	C4	CT	M	13.9330	8.3640	10.8900	-0.0660
52	51	H6	HC	E	13.0370	8.3820	11.2630	0.0340
53	51	H7	HC	E	14.3990	9.1620	11.1840	0.0340
54	51	C6	CT	M	13.8550	8.3460	9.3630	0.0320
55	54	H9	HC	E	14.7490	8.4150	8.9920	-0.0010
56	54	H10	HC	E	13.3440	9.1120	9.0570	-0.0010
57	54	C9	CT	M	13.1970	7.0660	8.8640	0.0320
58	57	H15	HC	E	13.2170	7.0480	7.8950	-0.0010
59	57	H16	HC	E	12.2690	7.0480	9.1470	-0.0010
60	57	C7	CT	M	13.9200	5.8420	9.4110	-0.0660
61	60	H11	HC	E	13.4650	5.0400	9.1090	0.0340

62	60	H12	HC	E	14.8250	5.8220	9.0630	0.0340

AJUYAFg

1	0	S1	SS	M	9.7790	0.9360	13.7720	-0.4160
2	1	C1	CM	M	11.4200	0.8850	14.1890	0.0080
3	2	N1	N	B	11.9850	-0.1280	14.8230	-0.3480
4	3	H1	H	E	11.5140	-0.8330	15.0660	0.2800
5	3	H2	H	E	12.8730	-0.1580	14.8890	0.2800
6	2	N2	N	M	12.2040	1.9370	13.8600	-0.0330
7	6	H3	H	E	11.9260	2.6130	13.4700	0.2290
8	6	N3	N	M	13.5680	1.9590	14.1500	-0.0330
9	8	H4	H	E	14.0450	1.3970	13.7390	0.2290
10	8	C2	CM	M	14.1420	2.9400	14.8680	0.0080
11	10	N4	N	B	13.3720	3.9410	15.2770	-0.3480
12	11	H5	H	E	13.7850	4.6480	15.6910	0.2800
13	11	H6	H	E	12.5250	3.9570	15.0630	0.2800
14	10	S2	SS	M	15.7960	2.8710	15.1960	-0.4160

BAFZENh

1	0	O11	O	M	6.3840	1.9050	4.5390	-0.5790
2	1	C15	C	M	6.8250	1.3290	5.5170	0.6250
3	2	O12	OH	S	7.2860	1.8860	6.5680	-0.6090
4	3	H22	HO	E	7.5140	1.2130	7.2140	0.3480
5	2	C13	CT	M	6.8080	-0.2070	5.5850	0.2000
6	5	H19	H1	E	7.8010	-0.5250	5.8630	0.0520
7	5	O9	OS	M	6.4640	-0.8050	4.3480	-0.4110
8	7	C11	CT	M	7.4890	-0.7630	3.3570	0.0920
9	8	H15	H1	E	7.5880	0.3700	3.6870	0.0330
10	8	H16	H1	E	8.3370	-1.3450	3.6870	0.0330
11	8	C9	CT	M	6.9530	-1.3320	2.0820	0.0330
12	11	H11	H1	E	6.5020	-2.2940	2.2790	0.0400
13	11	H12	H1	E	7.7590	-1.4500	1.3730	0.0400
14	11	O7	OS	M	5.9710	-0.4510	1.5460	-0.2870
15	14	C7	CT	M	5.3220	-1.0320	0.3900	0.0080
16	15	H7	H1	E	6.0640	-1.3330	-0.3350	0.0500
17	15	H8	H1	E	4.7420	-1.8910	0.6910	0.0500
18	15	C5	CT	M	4.4110	0.0120	-0.2130	0.1650
19	18	H3	H1	E	3.9200	-0.3880	-1.0880	0.0040
20	18	H4	H1	E	4.9850	0.8850	-0.4900	0.0040
21	18	O1	OS	M	3.4500	0.3570	0.7670	-0.3090
22	21	C1	CT	M	2.2030	0.8380	0.3090	0.1530
23	22	H1	H1	E	2.3240	1.2330	-0.6890	0.0480
24	22	C3	C	B	1.1590	-0.2640	0.2020	0.6020
25	24	O3	O	E	0.0000	0.0000	0.0000	-0.6930
26	24	O4	O	E	1.6370	-1.4680	0.3170	-0.6930
27	22	C2	CT	M	1.7390	1.9330	1.2720	0.2000
28	27	H2	H1	E	0.7750	2.3420	1.0070	0.0520
29	27	C4	C	B	2.7650	3.0770	1.2090	0.6250
30	29	O5	OH	S	2.7220	3.7680	0.1620	-0.6090
31	30	H21	HO	E	2.0040	3.4600	-0.3960	0.3480
32	29	O6	O	E	3.5600	3.2200	2.1240	-0.5790
33	27	O2	OS	M	1.6080	1.3640	2.5650	-0.4110
34	33	C6	CT	M	0.9760	2.2440	3.5020	0.0920
35	34	H5	H1	E	1.4850	3.1970	3.4950	0.0330
36	34	H6	H1	E	-0.0560	2.3850	3.2180	0.0330
37	34	C8	CT	M	1.0330	1.6640	4.8830	0.0330
38	37	H9	H1	E	0.5630	0.6920	4.8840	0.0400
39	37	H10	H1	E	0.5110	2.3170	5.5680	0.0400
40	37	O8	OS	M	2.3960	1.5350	5.2940	-0.2870
41	40	C10	CT	M	2.4960	1.1320	6.6650	0.0080
42	41	H13	H1	E	2.0790	1.9040	7.2950	0.0500
43	41	H14	H1	E	1.9470	0.2130	6.8090	0.0500
44	41	C12	CT	M	3.9320	0.9130	7.0370	0.1650
45	44	H17	H1	E	3.9990	0.6230	8.0740	0.0040
46	44	H18	H1	E	4.4920	1.8230	6.8810	0.0040
47	44	O10	OS	M	4.4630	-0.1280	6.2100	-0.3090
48	47	C14	CT	M	5.7320	-0.6370	6.5970	0.1530
49	48	H20	H1	E	5.9620	-0.2370	7.5740	0.0480
50	48	C16	C	M	5.7090	-2.1730	6.6690	0.6020
51	50	O14	O	E	6.7460	-2.7800	6.9030	-0.6930
52	50	O13	O	M	4.5680	-2.7200	6.5030	-0.6930

BAFZENg

1	0	N1	N3	M	1.7870	-3.1520	4.5380	-0.3350
2	1	H1	H	E	0.9750	-3.2160	5.2470	0.3540
3	1	H2	H	E	2.6120	-3.7570	4.8820	0.3540
4	1	H3	H	E	1.4540	-3.5090	3.5750	0.3540
5	1	C1	CT	M	2.2240	-1.7470	4.4190	-0.0190
6	5	H4	HP	E	1.3920	-1.1480	4.0810	0.1470
7	5	H5	HP	E	2.5500	-1.3970	5.3870	0.1470
8	5	C2	CT	M	3.3620	-1.6010	3.4380	-0.0190
9	8	H6	HP	E	3.0190	-1.8600	2.4470	0.1470
10	8	H7	HP	E	4.1680	-2.2600	3.7230	0.1470
11	8	N2	N3	M	3.8420	-0.2010	3.4430	-0.3350
12	11	H8	H	E	4.6570	-0.0970	2.7410	0.3540
13	11	H9	H	E	4.1860	0.0580	4.4330	0.3540
14	11	H10	H	E	3.0360	0.4570	3.1570	0.3540

BAPRAMh

1	0	O1	OS	M	7.8410	9.1120	0.0670	-0.3070
2	1	C1	CT	M	8.5310	8.7830	1.2770	0.0720
3	2	H1	H1	E	9.3870	9.2380	1.3030	0.0410
4	2	H2	H1	E	8.6920	7.8280	1.3150	0.0410
5	2	C2	CT	M	7.6960	9.2050	2.4450	0.0720
6	5	H3	H1	E	8.2050	9.1060	3.2650	0.0410
7	5	H4	H1	E	7.4460	10.1370	2.3530	0.0410
8	5	O2	OS	M	6.5270	8.3930	2.5010	-0.3070
9	8	C3	CT	M	5.6400	8.8230	3.5240	0.0720
10	9	H5	H1	E	5.3400	9.7270	3.3430	0.0410
11	9	H6	H1	E	6.0950	8.8170	4.3800	0.0410
12	9	C4	CT	M	4.4630	7.8940	3.5660	0.0720
13	12	H7	H1	E	4.7700	6.9830	3.6980	0.0410
14	12	H8	H1	E	3.8860	8.1290	4.3080	0.0410
15	12	O3	OS	M	3.7340	7.9860	2.3390	-0.3070
16	15	C5	CT	M	2.6820	7.0240	2.3150	0.0720
17	16	H9	H1	E	2.1210	7.1370	3.0970	0.0410
18	16	H10	H1	E	3.0580	6.1300	2.3390	0.0410
19	16	C7	CT	M	1.8550	7.1870	1.0750	0.0720
20	19	H23	H1	E	1.0480	6.6550	1.1510	0.0410
21	19	H24	H1	E	1.5960	8.1160	0.9760	0.0410
22	19	O4	OS	M	2.5960	6.7720	-0.0670	-0.3070
23	22	C8	CT	M	1.9060	7.1010	-1.2770	0.0720
24	23	H13	H1	E	1.0500	6.6460	-1.3030	0.0410
25	23	H14	H1	E	1.7450	8.0560	-1.3150	0.0410
26	23	C9	CT	M	2.7410	6.6790	-2.4450	0.0720
27	26	H15	H1	E	2.2320	6.7780	-3.2650	0.0410
28	26	H16	H1	E	2.9910	5.7470	-2.3530	0.0410
29	26	O5	OS	M	3.9100	7.4910	-2.5010	-0.3070
30	29	C10	CT	M	4.7970	7.0610	-3.5240	0.0720
31	30	H17	H1	E	5.0970	6.1570	-3.3430	0.0410
32	30	H18	H1	E	4.3420	7.0670	-4.3800	0.0410
33	30	C11	CT	M	5.9740	7.9900	-3.5660	0.0720
34	33	H19	H1	E	5.6670	8.9010	-3.6980	0.0410
35	33	H20	H1	E	6.5510	7.7550	-4.3080	0.0410
36	33	O6	OS	M	6.7030	7.8980	-2.3390	-0.3070
37	36	C12	CT	M	7.7550	8.8600	-2.3150	0.0720
38	37	H21	H1	E	8.3160	8.7470	-3.0970	0.0410
39	37	H22	H1	E	7.3790	9.7540	-2.3390	0.0410
40	37	C6	CT	M	8.5820	8.6970	-1.0750	0.0720
41	40	H11	H1	E	9.3890	9.2290	-1.1510	0.0410
42	40	H12	H1	E	8.8410	7.7680	-0.9760	0.0410

BAPRAMg

1	0	S1	SS	M	6.0350	4.6750	2.4590	-0.4480
2	1	C8	CM	M	6.3840	5.1210	0.8470	0.1320
3	2	N3	NT	B	6.2200	6.3440	0.3870	-0.3450
4	3	H3	H	E	6.3840	6.5440	-0.4570	0.2630
5	3	H4	H	E	6.0180	6.9410	0.9320	0.2630
6	2	N2	NT	M	6.8300	4.1770	0.0030	-0.3130
7	6	H2	H	E	6.9680	3.4070	0.2670	0.3050
8	6	N1	N	M	7.2540	4.4840	-1.2830	-0.1880

9	8	H1	H	E	6.7500	4.2170	-1.8660	0.2600
10	8	C7	C	M	8.6060	4.6440	-1.4930	0.6110
11	10	O1	O	E	9.3650	4.7500	-0.5410	-0.4890
12	10	C1	CA	M	9.0450	4.7020	-2.9020	-0.1840
13	12	C2	CA	M	8.1800	5.1460	-3.9110	-0.1480
14	13	H7	HA	E	7.3070	5.3730	-3.6880	0.1020
15	13	C3	CA	M	8.5820	5.2560	-5.2220	-0.1470
16	15	H8	HA	E	7.9940	5.5550	-5.8770	0.1430
17	15	C4	CA	M	9.8900	4.9070	-5.5450	-0.1620
18	17	H9	HA	E	10.1810	4.9870	-6.4260	0.1650
19	17	C5	CA	M	10.7580	4.4490	-4.5840	-0.1680
20	19	H10	HA	E	11.6230	4.2140	-4.8310	0.1400
21	19	C6	CA	M	10.3730	4.3250	-3.2360	0.2890
22	21	N4	NH	M	11.2450	3.8080	-2.3330	-0.8620
23	22	H5	H	E	11.9900	3.6220	-2.5900	0.3900
24	22	H6	H	E	11.0960	3.8040	-1.4750	0.3900

BAPREQh

1	0	O1	OS	M	1.8450	0.2580	-1.0180	-0.3180
2	1	C1	CT	M	1.2410	-0.9900	-1.3610	0.0850
3	2	H1	H1	E	1.7940	-1.4560	-2.0080	0.0370
4	2	H2	H1	E	0.3720	-0.8360	-1.7600	0.0370
5	2	C2	CT	M	1.0900	-1.8320	-0.1160	0.0850
6	5	H3	H1	E	0.5220	-1.3560	0.5100	0.0370
7	5	H4	H1	E	0.6370	-2.6560	-0.3540	0.0370
8	5	O2	OS	M	2.3150	-2.1610	0.5420	-0.3180
9	8	C3	CT	M	3.2240	-2.9140	-0.2570	0.0850
10	9	H5	H1	E	3.5060	-2.3920	-1.0230	0.0370
11	9	H6	H1	E	2.7940	-3.7230	-0.5780	0.0370
12	9	C4	CT	M	4.4090	-3.2630	0.5940	0.0850
13	12	H7	H1	E	4.1200	-3.7410	1.3880	0.0370
14	12	H8	H1	E	5.0190	-3.8320	0.0990	0.0370
15	12	O3	OS	M	5.0610	-2.0480	0.9640	-0.3180
16	15	C5	CT	M	6.2240	-2.2720	1.7570	0.0850
17	16	H9	H1	E	5.9960	-2.7920	2.5430	0.0370
18	16	H10	H1	E	6.8850	-2.7650	1.2450	0.0370
19	16	C7	CT	M	6.7800	-0.9380	2.1660	0.0850
20	19	H23	H1	E	7.5000	-1.0640	2.8040	0.0370
21	19	H24	H1	E	6.0860	-0.4100	2.5920	0.0370
22	19	O4	OS	M	7.2670	-0.2580	1.0180	-0.3180
23	22	C8	CT	M	7.8710	0.9900	1.3610	0.0850
24	23	H13	H1	E	7.3180	1.4560	2.0080	0.0370
25	23	H14	H1	E	8.7400	0.8360	1.7600	0.0370
26	23	C9	CT	M	8.0220	1.8320	0.1160	0.0850
27	26	H15	H1	E	8.5900	1.3560	-0.5100	0.0370
28	26	H16	H1	E	8.4750	2.6560	0.3540	0.0370
29	26	O5	OS	M	6.7970	2.1610	-0.5420	-0.3180
30	29	C10	CT	M	5.8880	2.9140	0.2570	0.0850
31	30	H17	H1	E	5.6060	2.3920	1.0230	0.0370
32	30	H18	H1	E	6.3180	3.7230	0.5780	0.0370
33	30	C11	CT	M	4.7030	3.2630	-0.5940	0.0850
34	33	H19	H1	E	4.9920	3.7410	-1.3880	0.0370
35	33	H20	H1	E	4.0930	3.8320	-0.0990	0.0370
36	33	O6	OS	M	4.0510	2.0480	-0.9640	-0.3180
37	36	C12	CT	M	2.8880	2.2720	-1.7570	0.0850
38	37	H21	H1	E	3.1160	2.7920	-2.5430	0.0370
39	37	H22	H1	E	2.2270	2.7650	-1.2450	0.0370
40	37	C6	CT	M	2.3320	0.9380	-2.1660	0.0850
41	40	H11	H1	E	1.6120	1.0640	-2.8040	0.0370
42	40	H12	H1	E	3.0260	0.4100	-2.5920	0.0370

BAPREQg

1	0	S1	SS	M	2.6280	2.8620	2.6090	-0.4270
2	1	C4	C	M	2.9750	1.1970	2.6310	0.0450
3	2	N3	N	B	3.3090	0.5270	1.5530	-0.3050
4	3	H3	H	E	3.3920	-0.3070	1.5750	0.2600
5	3	H4	H	E	3.3080	0.9310	0.7920	0.2600
6	2	N2	N	M	2.9170	0.5370	3.8110	-0.1820
7	6	H2	H	E	2.6120	0.9370	4.5060	0.2780
8	6	N1	N	M	3.0290	-0.8430	3.8570	-0.1620
9	8	H1	H	E	3.7290	-1.1560	4.1540	0.2150

10	8	C3	C	M	1.9440	-1.6280	3.6160	0.4940
11	10	O1	O	E	0.8580	-1.1850	3.2830	-0.4730
12	10	C1	CV	M	2.1830	-3.0810	3.7720	0.1040
13	12	N5	NB	M	3.3410	-3.5740	4.1990	-0.3710
14	13	S2	S	M	3.1830	-5.1980	4.2620	0.2750
15	14	N6	NB	M	1.6400	-5.3200	3.7460	-0.4640
16	15	C2	CR	M	1.1960	-4.0870	3.4970	0.5740
17	16	N4	NH	M	-0.0290	-3.8460	2.9960	-0.9390
18	17	H5	H	E	-0.6090	-4.4820	2.9780	0.4090
19	17	H6	H	E	-0.2930	-2.9950	2.9310	0.4090

BEGVOZh

1	0	O1	OS	M	-1.1100	1.1860	-2.0620	-0.3650
2	1	C1	CT	M	-1.0500	2.5400	-2.4690	0.0290
3	2	H1	H1	E	-0.3690	2.9930	-1.9480	0.0900
4	2	H2	H1	E	-1.9000	2.9630	-2.2730	0.0900
5	2	C2	CT	M	-0.7440	2.7230	-3.9320	-0.0520
6	5	H3	HP	E	-1.5090	2.4390	-4.4570	0.1200
7	5	H4	HP	E	-0.5920	3.6640	-4.1130	0.1200
8	5	N1	N3	M	0.4540	1.9440	-4.3470	0.0030
9	8	H5	H	E	1.0950	1.9920	-3.7100	0.2340
10	8	H6	H	E	0.1800	1.0890	-4.4620	0.2340
11	8	C3	CT	M	1.0420	2.4240	-5.6240	-0.0520
12	11	H7	HP	E	1.3610	3.3330	-5.5080	0.1200
13	11	H8	HP	E	0.3560	2.4330	-6.3100	0.1200
14	11	C4	CT	M	2.1850	1.5510	-6.0760	0.0290
15	14	H9	H1	E	1.9050	0.6220	-6.0960	0.0900
16	14	H10	H1	E	2.4640	1.8060	-6.9690	0.0900
17	14	O2	OS	M	3.2690	1.7170	-5.1580	-0.3650
18	17	C5	CT	M	4.3670	0.8130	-5.4080	-0.0160
19	18	H11	H1	E	5.1980	1.2570	-5.1780	0.1240
20	18	H12	H1	E	4.3950	0.6090	-6.3550	0.1240
21	18	C7	CT	M	4.2720	-0.4630	-4.6400	-0.0160
22	21	H27	H1	E	4.1850	-0.2740	-3.6920	0.1240
23	21	H28	H1	E	5.0740	-0.9920	-4.7750	0.1240
24	21	O3	OS	M	3.1260	-1.1860	-5.1010	-0.3650
25	24	C8	CT	M	3.0660	-2.5400	-4.6940	0.0290
26	25	H15	H1	E	2.3850	-2.9930	-5.2140	0.0900
27	25	H16	H1	E	3.9160	-2.9630	-4.8900	0.0900
28	25	C9	CT	M	2.7600	-2.7230	-3.2300	-0.0520
29	28	H17	HP	E	2.6080	-3.6640	-3.0500	0.1200
30	28	H18	HP	E	3.5250	-2.4390	-2.7050	0.1200
31	28	N2	N3	M	1.5630	-1.9440	-2.8160	0.0030
32	31	H19	H	E	0.9210	-1.9920	-3.4520	0.2340
33	31	H20	H	E	1.8360	-1.0890	-2.7000	0.2340
34	31	C10	CT	M	0.9740	-2.4240	-1.5390	-0.0520
35	34	H21	HP	E	0.6550	-3.3330	-1.6550	0.1200
36	34	H22	HP	E	1.6600	-2.4330	-0.8530	0.1200
37	34	C11	CT	M	-0.1690	-1.5510	-1.0870	0.0290
38	37	H23	H1	E	0.1110	-0.6220	-1.0670	0.0900
39	37	H24	H1	E	-0.4480	-1.8060	-0.1930	0.0900
40	37	O4	OS	M	-1.2530	-1.7170	-2.0050	-0.3650
41	40	C12	CT	M	-2.3510	-0.8130	-1.7550	-0.0160
42	41	H25	H1	E	-3.1820	-1.2570	-1.9850	0.1240
43	41	H26	H1	E	-2.3790	-0.6090	-0.8070	0.1240
44	41	C6	CT	M	-2.2560	0.4630	-2.5230	-0.0160
45	44	H13	H1	E	-2.1690	0.2740	-3.4700	0.1240
46	44	H14	H1	E	-3.0580	0.9920	-2.3880	0.1240

BEGVOZg

1	0	O4	O	M	2.6170	0.4090	-1.8820	-0.3720
2	1	N4	N2	M	2.3570	1.6320	-2.1960	-0.1970
3	2	C1	CM	M	3.1190	2.5900	-1.7030	0.3270
4	3	C2	C	M	2.7030	3.9340	-2.0880	0.2070
5	4	O1	O	E	1.7930	4.1940	-2.8640	-0.5870
6	4	N1	N	M	3.3990	4.9750	-1.5010	-0.2440
7	6	H2	H	E	3.1080	5.7610	-1.6040	0.2960
8	6	C3	C	M	4.4490	4.8700	-0.6240	0.3450
9	8	O2	O	E	4.9800	5.8410	-0.1330	-0.5840
10	8	N2	N	M	4.8560	3.5980	-0.3600	-0.1670
11	10	H3	H	E	5.4980	3.5110	0.1580	0.2790

12	10	C4	CM	M	4.2810	2.4340	-0.8300	0.1940
13	12	N3	N2	M	4.7350	1.2830	-0.4940	-0.3770
14	13	O3	OH	M	5.8370	1.4180	0.3800	-0.5620
15	14	H1	HO	E	5.9120	0.4970	0.6370	0.4420

CECMEC10h

1	0	C9	CT	M	-2.8580	7.2210	3.7660	0.0030
2	1	H13	H1	E	-1.9790	7.3590	3.5280	0.0550
3	1	H64	H1	E	-3.2380	6.4100	4.4000	0.0550
4	1	H82	H1	E	-3.3700	7.1690	2.6560	0.0550
5	1	O5	OS	M	-3.2650	8.4870	4.2700	-0.3000
6	5	C6	CT	M	-2.6750	8.7830	5.5490	-0.0170
7	6	H6	H1	E	-3.2330	9.4720	5.9860	0.0860
8	6	H7	H1	E	-2.6720	7.8810	6.1310	0.0860
9	6	C5	CT	M	-1.2380	9.2940	5.4060	0.0200
10	9	O4	OS	E	-1.3460	10.6070	4.7910	-0.3150
11	9	H5	H1	E	-0.6630	8.6650	4.8100	0.1480
12	9	C4	CT	M	-0.6000	9.4270	6.7840	-0.0070
13	12	O3	OS	E	-0.2240	8.1280	7.2370	-0.2360
14	12	H4	H1	E	-1.3100	9.8520	7.4920	0.1440
15	12	C3	CT	M	0.6440	10.3100	6.7930	-0.0350
16	15	O2	OS	S	0.8160	10.6920	8.1700	-0.3450
17	16	C8	CT	3	2.1100	11.0200	8.5770	-0.0150
18	17	H12	H1	E	2.7510	11.8460	8.4170	0.0650
19	17	H63	H1	E	3.0130	10.5880	7.8490	0.0650
20	17	H81	H1	E	2.4750	10.7060	9.6600	0.0650
21	15	H3	H1	E	1.4460	9.8750	6.3960	0.1660
22	15	C2	CT	M	0.5390	11.5210	5.9110	0.0310
23	22	O1	OS	S	1.8620	12.0550	5.7810	-0.2340
24	23	C7	CT	3	1.8960	13.4430	5.4830	-0.1270
25	24	H11	H1	E	0.9400	13.8400	5.9860	0.0890
26	24	H62	H1	E	1.4110	13.5790	4.4800	0.0890
27	24	H80	H1	E	2.7440	13.5550	5.3520	0.0890
28	22	H2	H1	E	-0.1080	12.1780	6.4220	0.1470
29	22	C1	CT	M	-0.0890	11.2190	4.5360	0.0060
30	29	H1	H2	E	-0.2490	12.0830	3.9250	0.1690
31	29	O8	OS	M	0.7440	10.4140	3.7570	-0.2360
32	31	C13	CT	M	0.7880	10.6900	2.3530	-0.0070
33	32	C14	CT	3	-0.1730	9.7800	1.5780	0.0200
34	33	O9	OS	E	0.1510	9.7190	0.1760	-0.3150
35	33	C15	CT	3	-1.5930	10.2880	1.6160	-0.0170
36	35	O10	OS	S	-2.4320	9.3700	0.9670	-0.3000
37	36	C18	CT	3	-3.7900	9.8230	0.9580	0.0030
38	37	H16	H1	E	-4.2440	9.2580	0.3960	0.0550
39	37	H67	H1	E	-4.0850	9.9700	2.1270	0.0550
40	37	H85	H1	E	-3.8040	10.6590	0.7930	0.0550
41	35	H33	H1	E	-1.9020	10.3740	2.6430	0.0860
42	35	H34	H1	E	-1.6670	11.2520	1.1100	0.0860
43	33	H32	H1	E	-0.0620	8.7360	1.9690	0.1480
44	32	H31	H1	E	0.4330	11.6560	2.1010	0.1440
45	32	C12	CT	M	2.2720	10.4550	1.9490	-0.0350
46	45	O7	OS	S	3.0120	11.5470	2.4590	-0.3450
47	46	C17	CT	3	4.1200	11.1690	3.2650	-0.0150
48	47	H15	H1	E	4.6310	10.4690	2.6030	0.0650
49	47	H66	H1	E	3.5170	11.0860	3.9640	0.0650
50	47	H84	H1	E	4.3430	11.8930	3.7660	0.0650
51	45	H30	H1	E	2.6830	9.6380	2.3920	0.1660
52	45	C11	CT	M	2.3880	10.3980	0.4480	0.0310
53	52	O6	OS	S	3.7560	10.1340	0.0910	-0.2340
54	53	C16	CT	3	4.2630	11.0550	-0.8140	-0.1270
55	54	H14	H1	E	4.2940	12.0410	-0.3510	0.0890
56	54	H65	H1	E	3.6240	11.0880	-1.6970	0.0890
57	54	H83	H1	E	5.2710	10.7600	-1.1060	0.0890
58	52	H9	H1	E	2.0960	11.3000	0.0260	0.1470
59	52	C10	CT	M	1.4930	9.3390	-0.1150	0.0060
60	59	H8	H2	E	1.5780	9.2340	-1.1630	0.1690
61	59	O13	OS	M	1.7820	8.0830	0.4990	-0.2360
62	61	C22	CT	M	1.9680	6.9370	-0.3360	-0.0070
63	62	C23	CT	3	0.7030	6.0910	-0.4070	0.0200
64	63	O14	OS	E	0.9960	4.8690	-1.1340	-0.3150
65	63	C24	CT	3	-0.3870	6.7750	-1.2160	-0.0170
66	65	O15	OS	S	-1.5870	6.0130	-1.0600	-0.3000

67	66	C27	CT	3	-2.7650	6.6870	-1.2920	0.0030
68	67	H19	H1	E	-3.5250	6.3400	-0.5920	0.0550
69	67	H70	H1	E	-2.6060	7.7560	-1.1540	0.0550
70	67	H88	H1	E	-3.0980	6.4970	-2.3120	0.0550
71	65	H40	H1	E	-0.5620	7.8340	-0.8460	0.0860
72	65	H41	H1	E	-0.0950	6.8130	-2.3390	0.0860
73	63	H39	H1	E	0.3690	5.8400	0.7140	0.1480
74	62	H38	H1	E	2.2380	7.2400	-1.3870	0.1440
75	62	C21	CT	M	3.0910	6.1200	0.3400	-0.0350
76	75	O12	OS	S	4.3220	6.8440	0.2660	-0.3450
77	76	C26	CT	3	4.8520	7.1690	1.5500	-0.0150
78	77	H18	H1	E	4.7450	6.1720	2.2600	0.0650
79	77	H69	H1	E	3.9550	7.6440	2.2200	0.0650
80	77	H87	H1	E	5.5650	7.1690	1.6910	0.0650
81	75	H37	H1	E	2.8300	6.0060	1.4270	0.1660
82	75	C20	CT	M	3.2720	4.7910	-0.4060	0.0310
83	82	O11	OS	S	4.2200	3.9930	0.3030	-0.2340
84	83	C25	CT	3	5.4780	3.8480	-0.3580	-0.1270
85	84	H17	H1	E	6.0970	3.0390	0.3700	0.0890
86	84	H68	H1	E	6.1260	4.7070	-0.5310	0.0890
87	84	H86	H1	E	5.0890	3.3710	-1.3610	0.0890
88	82	H36	H1	E	3.7410	4.9380	-1.4400	0.1470
89	82	C19	CT	M	1.9560	4.0330	-0.5020	0.0060
90	89	H35	H2	E	1.9810	3.0390	-1.1500	0.1690
91	89	O18	OS	M	1.5200	3.6130	0.7690	-0.2360
92	91	C31	CT	M	0.9070	2.3070	0.8420	-0.0070
93	92	C32	CT	3	-0.4920	2.4880	1.4600	0.0200
94	93	O19	OS	E	-1.0240	1.2010	1.8620	-0.3150
95	93	C33	CT	3	-1.4980	3.0740	0.5190	-0.0170
96	95	O20	OS	S	-1.5970	2.3030	-0.6610	-0.3000
97	96	C36	CT	3	-2.7380	2.6940	-1.4600	0.0030
98	97	H22	H1	E	-2.5830	1.8040	-2.2600	0.0550
99	97	H73	H1	E	-3.5880	2.6350	-0.9510	0.0550
100	97	H91	H1	E	-2.7960	3.7980	-1.3870	0.0550
101	95	H47	H1	E	-1.2950	4.1070	0.2510	0.0860
102	95	H48	H1	E	-2.5070	3.0860	1.0040	0.0860
103	93	H46	H1	E	-0.4200	3.0620	2.3520	0.1480
104	92	H45	H1	E	0.8070	1.8990	-0.1720	0.1440
105	92	C30	CT	M	1.8070	1.4390	1.7260	-0.0350
106	105	O17	OS	S	2.9540	1.1160	0.9220	-0.3450
107	106	C35	CT	3	4.3080	1.1130	1.4140	-0.0150
108	107	H21	H1	E	5.0440	0.8310	0.6610	0.0650
109	107	H72	H1	E	4.2570	0.4750	2.1800	0.0650
110	107	H90	H1	E	4.6100	2.1060	1.7470	0.0650
111	105	H44	H1	E	2.1630	1.9470	2.6560	0.1660
112	105	C29	CT	M	1.0930	0.1780	2.1820	0.0310
113	112	O16	OS	S	1.9370	-0.5130	3.0830	-0.2340
114	113	C34	CT	3	2.0660	-1.8750	2.8370	-0.1270
115	114	H20	H1	E	2.3720	-2.3500	3.5550	0.0890
116	114	H71	H1	E	1.3060	-1.9940	3.5810	0.0890
117	114	H89	H1	E	1.2500	-2.3500	2.2070	0.0890
118	112	H43	H1	E	0.8780	-0.5220	1.3480	0.1470
119	112	C28	CT	M	-0.2310	0.5340	2.8240	0.0060
120	119	H42	H2	E	-0.7880	-0.3320	3.0790	0.1690
121	119	O23	OS	M	0.0190	1.3670	3.9470	-0.2360
122	121	C40	CT	M	-0.7850	1.2110	5.1350	-0.0070
123	122	C41	CT	3	-1.7510	2.4000	5.2740	0.0200
124	123	O24	OS	E	-2.3230	2.4050	6.5830	-0.3150
125	123	C42	CT	3	-2.9350	2.2960	4.3100	-0.0170
126	125	O25	OS	S	-3.5770	3.5660	4.3090	-0.3000
127	126	C45	CT	3	-4.7690	3.4830	3.5630	0.0030
128	127	H27	H1	E	-4.6980	3.2290	2.3920	0.0550
129	127	H76	H1	E	-5.2430	2.6830	3.9510	0.0550
130	127	H94	H1	E	-5.1250	4.7480	3.3560	0.0550
131	125	H24	H1	E	-3.6810	1.5430	4.6780	0.0860
132	125	H53	H1	E	-2.7140	2.1130	3.3040	0.0860
133	123	H52	H1	E	-1.2220	3.3710	5.1670	0.1480
134	122	H51	H1	E	-1.3690	0.3320	5.0350	0.1440
135	122	C39	CT	M	0.1970	1.1630	6.2990	-0.0350
136	135	O22	OS	S	0.8180	-0.1420	6.2480	-0.3450
137	136	C44	CT	3	2.2880	-0.0620	6.2750	-0.0150
138	137	H26	H1	E	2.8960	0.2920	5.4420	0.0650
139	137	H75	H1	E	2.3990	-1.1630	6.4750	0.0650

140	137	H93	H1	E	2.6100	0.6880	7.0170	0.0650
141	135	H50	H1	E	0.9430	1.9700	6.2110	0.1660
142	135	C38	CT	M	-0.4890	1.3150	7.6260	0.0310
143	142	O21	OS	S	0.5280	1.4030	8.6260	-0.2340
144	143	C43	CT	3	0.0980	1.2040	9.9380	-0.1270
145	144	H25	H1	E	-0.5880	1.7090	10.0690	0.0890
146	144	H74	H1	E	0.1720	0.1190	9.9370	0.0890
147	144	H92	H1	E	0.7160	1.1630	10.5710	0.0890
148	142	H23	H1	E	-1.1350	0.4620	7.8320	0.1470
149	142	C37	CT	M	-1.3740	2.5470	7.6220	0.0060
150	149	H49	H2	E	-1.8720	2.7300	8.4840	0.1690
151	149	O28	OS	M	-0.6030	3.7220	7.4620	-0.2360
152	151	C49	CT	M	-0.8590	4.8380	8.2990	-0.0070
153	152	H57	H1	E	-1.2740	4.4630	9.2100	0.1440
154	152	C48	CT	M	0.4790	5.4880	8.5380	-0.0350
155	154	O27	OS	S	1.2500	4.6720	9.4120	-0.3450
156	155	C53	CT	3	2.4460	4.1570	8.8930	-0.0150
157	156	H29	H1	E	3.0000	4.9850	8.2060	0.0650
158	156	H78	H1	E	2.2300	3.9640	7.9420	0.0650
159	156	H96	H1	E	2.8610	3.5610	9.9240	0.0650
160	154	H56	H1	E	0.9720	5.6020	7.5590	0.1660
161	154	C47	CT	M	0.3150	6.8720	9.2140	0.0310
162	161	O26	OS	S	1.5800	7.5160	9.2640	-0.2340
163	162	C52	CT	3	1.9530	7.8910	10.5290	-0.1270
164	163	H28	H1	E	1.6070	7.5960	11.4830	0.0890
165	163	H77	H1	E	1.1050	8.3100	11.0700	0.0890
166	163	H95	H1	E	2.7740	8.2610	10.3730	0.0890
167	161	H55	H1	E	-0.0460	6.7890	10.2810	0.1470
168	161	C46	CT	M	-0.7000	7.7480	8.5300	0.0060
169	168	H54	H2	E	-0.8480	8.6650	9.1440	0.1690
170	168	O29	OS	M	-1.9220	7.0340	8.4150	-0.3150
171	170	C50	CT	M	-1.8130	5.8260	7.6300	0.0200
172	171	H58	H1	E	-1.3680	6.0300	6.6340	0.1480
173	171	C51	CT	M	-3.2550	5.3220	7.4280	-0.0170
174	173	H59	H1	E	-3.8530	6.1010	7.0170	0.0860
175	173	H60	H1	E	-3.2360	4.4630	6.6600	0.0860
176	173	O30	OS	M	-3.7740	4.9120	8.7120	-0.3000
177	176	C54	CT	M	-5.1580	5.0710	8.7990	0.0030
178	177	H10	H1	E	-5.5720	6.0060	8.3780	0.0550
179	177	H61	H1	E	-5.7380	4.7720	9.2500	0.0550
180	177	H79	H1	E	-5.5620	4.3440	7.9550	0.0550

CECMEC10g

1	0	O2	OH	M	5.6440	8.4530	4.6570	-0.6900
2	1	H8	HO	E	6.8750	8.4270	4.3210	0.4930
3	1	C7	C	M	5.4050	8.0620	5.8510	0.6970
4	3	O3	O	E	6.2360	7.8860	6.7420	-0.5760
5	3	C4	CT	M	3.8830	7.9740	6.2010	0.2270
6	5	O1	OH	S	3.8050	7.8270	7.5890	-0.6540
7	6	H6	HO	E	2.6450	7.5490	7.9550	0.4230
8	5	H3	H1	E	3.4320	8.9970	5.8140	0.0640
9	5	C1	CA	M	3.2080	6.8650	5.4460	0.0760
10	9	C2	CA	M	2.0960	7.1240	4.6200	-0.1430
11	10	H1	HA	E	1.7020	8.1900	4.3610	0.1300
12	10	C5	CA	M	1.4410	6.1010	3.9850	-0.1760
13	12	H4	HA	E	0.6380	6.4570	3.2110	0.1470
14	12	C8	CA	M	1.8580	4.8000	4.1740	-0.1100
15	14	H7	HA	E	1.2710	3.9410	3.7000	0.1330
16	14	C6	CA	M	2.9570	4.5130	4.9610	-0.1760
17	16	H5	HA	E	3.3960	3.3230	5.0740	0.1470
18	16	C3	CA	M	3.6320	5.5670	5.5790	-0.1430
19	18	H2	HA	E	4.4770	5.4120	6.2500	0.1300

DESHEOh

1	0	O5	OH	M	9.7430	17.8280	6.9930	-0.5250
2	1	H5	HO	E	10.5450	17.0900	6.9710	0.3990
3	1	C7	CA	M	8.6710	17.3130	6.4940	0.4290
4	3	C6	CA	S	7.4820	18.0580	6.4040	-0.3690
5	4	H2	HA	E	7.4780	19.0980	6.7300	0.1950
6	3	C4	CA	M	8.5440	15.9840	6.0310	-0.3690
7	6	H1	HA	E	9.3990	15.3100	6.0840	0.1950

8	6	C1	CA	M	7.3530	15.5330	5.5150	0.2800
9	8	N1	NC	M	6.2130	16.2400	5.4480	-0.5810
10	9	C2	CA	M	6.3180	17.4980	5.9100	0.2800
11	10	C5	C	M	5.0990	18.3710	5.8890	0.7660
12	11	O4	O	E	5.1110	19.4960	6.1990	-0.6060
13	11	O3	OS	M	4.0010	17.7450	5.5330	-0.3810
14	13	C9	CT	M	2.7480	18.4830	5.5700	0.1360
15	14	H6	H1	E	2.9370	19.4990	5.9170	0.0580
16	14	H7	H1	E	2.0570	17.9890	6.2530	0.0580
17	14	C11	CT	M	2.1580	18.5280	4.2270	0.0630
18	17	H10	H1	E	2.8720	18.9750	3.5350	0.0570
19	17	H11	H1	E	1.2510	19.1320	4.2520	0.0570
20	17	O7	OS	M	1.8380	17.2230	3.7890	-0.3780
21	20	C13	CT	M	1.2180	17.2090	2.5150	0.1020
22	21	H14	H1	E	1.8480	17.7330	1.7970	0.0500
23	21	H15	H1	E	0.2480	17.7020	2.5760	0.0500
24	21	C15	CT	M	1.0340	15.8030	2.0810	0.0930
25	24	H18	H1	E	0.5210	15.7910	1.1200	0.0510
26	24	H19	H1	E	0.4300	15.2770	2.8200	0.0510
27	24	O8	OS	M	2.2610	15.1550	1.9500	-0.3910
28	27	C14	CT	M	2.1400	13.8410	1.4860	0.0930
29	28	H16	H1	E	1.6210	13.2390	2.2320	0.0510
30	28	H17	H1	E	1.5720	13.8360	0.5560	0.0510
31	28	C12	CT	M	3.4790	13.2760	1.2460	0.1020
32	31	H12	H1	E	3.3830	12.3050	0.7610	0.0500
33	31	H13	H1	E	4.0480	13.9480	0.6030	0.0500
34	31	O6	OS	M	4.1660	13.1190	2.5090	-0.3780
35	34	C10	CT	M	5.4840	12.6500	2.3180	0.0630
36	35	H8	H1	E	5.4500	11.6610	1.8610	0.0570
37	35	H9	H1	E	6.0170	13.3350	1.6590	0.0570
38	35	C8	CT	M	6.1960	12.5640	3.6090	0.1360
39	38	H3	H1	E	5.6240	11.9500	4.3050	0.0580
40	38	H4	H1	E	7.1810	12.1220	3.4570	0.0580
41	38	O2	OS	M	6.3390	13.9070	4.1430	-0.3810
42	41	C3	C	M	7.3300	14.1110	5.0150	0.7660
43	42	O1	O	M	8.1160	13.2710	5.3330	-0.6060

DESHEOg

1	0	N1	N3	M	3.6950	15.1300	4.5030	-0.3210
2	1	H1	H	E	3.4210	15.7350	4.0060	0.3140
3	1	H2	H	E	4.5030	15.3550	4.8020	0.3140
4	1	H3	H	E	3.7910	14.5480	3.8850	0.3140
5	1	C1	CT	M	2.8370	14.6550	5.6050	0.0130
6	5	H4	HP	E	1.9660	14.4490	5.2550	0.1200
7	5	H5	HP	E	2.7620	15.3500	6.2620	0.1200
8	5	C2	CA	M	3.3980	13.4380	6.2500	-0.0140
9	8	C3	CA	M	2.7390	12.2340	6.1680	-0.1590
10	9	H6	HA	E	1.9330	12.1720	5.6510	0.1570
11	9	C5	CA	M	3.2150	11.1230	6.8120	-0.1450
12	11	H8	HA	E	2.7410	10.2900	6.7450	0.1720
13	11	C7	CA	M	4.3670	11.1940	7.5500	-0.0670
14	13	H10	HA	E	4.6940	10.4140	8.0070	0.1610
15	13	C6	CA	M	5.0490	12.3740	7.6360	-0.1450
16	15	H9	HA	E	5.8650	12.4260	8.1370	0.1720
17	15	C4	CA	M	4.5520	13.5010	6.9920	-0.1590
18	17	H7	HA	E	5.0230	14.3340	7.0680	0.1570

DOXWAOh

1	0	O9	OS	M	-2.7560	9.2080	10.4180	-0.3020
2	1	C21	CT	M	-1.9120	9.7510	9.3220	0.0680
3	2	H29	H1	E	-1.4250	8.9570	8.7920	0.0600
4	2	H30	H1	E	-1.1770	10.4240	9.7070	0.0600
5	2	C19	CT	M	-2.8560	10.4580	8.4430	0.0670
6	5	H25	H1	E	-2.3140	10.9050	7.6330	0.0400
7	5	H26	H1	E	-3.3490	11.2260	9.0040	0.0400
8	5	O7	OS	M	-3.7950	9.5960	7.9360	-0.2760
9	8	C16	CT	M	-4.4970	9.8890	6.8450	0.0210
10	9	H18	H1	E	-4.3670	10.9420	6.7290	0.0610
11	9	H19	H1	E	-3.9720	9.3780	6.0580	0.0610
12	9	C14	CT	M	-5.6770	9.7080	6.5790	0.0060
13	12	H14	H1	E	-5.7250	9.4580	5.5330	0.0970

14	12	H15	H1	E	-6.1820	10.6310	6.7450	0.0970
15	12	O4	OS	M	-6.3750	8.7340	7.2530	-0.3440
16	15	C11	CT	M	-7.8030	8.7510	7.0560	0.0060
17	16	H11	H1	E	-8.1440	9.7600	7.1620	0.1300
18	16	C9	CT	M	-8.1980	8.2770	5.6540	0.0450
19	18	H9	H1	E	-7.6900	7.3500	5.4980	0.1140
20	18	O2	OS	M	-7.8360	9.2260	4.6260	-0.2670
21	20	C4	CT	M	-8.2400	8.7430	3.3410	0.1730
22	21	C1	CA	S	-7.7500	9.7770	2.3020	0.0600
23	22	C2	CA	B	-6.5760	10.4930	2.5200	-0.1180
24	23	C5	CA	B	-6.1880	11.4850	1.6160	-0.1680
25	24	C7	CA	B	-6.9730	11.7520	0.4960	-0.1200
26	25	C6	CA	B	-8.1460	11.0280	0.2780	-0.1680
27	26	C3	CA	S	-8.5350	10.0450	1.1800	-0.1180
28	27	H2	HA	E	-9.4340	9.4890	1.0130	0.1220
29	26	H5	HA	E	-8.7500	11.2370	-0.5690	0.1370
30	25	H6	HA	E	-6.6740	12.5110	-0.1970	0.1280
31	24	H4	HA	E	-5.2850	12.0370	1.7840	0.1370
32	23	H1	HA	E	-5.9710	10.2860	3.3780	0.1220
33	21	H3	H2	E	-7.8240	7.7640	3.2150	0.0890
34	21	O1	OS	M	-9.5870	8.6220	3.1980	-0.3420
35	34	C8	CT	M	-10.1500	7.6910	4.1840	-0.0180
36	35	H7	H1	E	-9.7810	6.7010	4.0020	0.0950
37	35	H8	H1	E	-11.2170	7.6900	4.1170	0.0950
38	35	C10	CT	M	-9.7120	8.1650	5.5690	0.0970
39	38	H10	H1	E	-10.1290	9.1290	5.7570	0.1470
40	38	O3	OS	M	-10.1750	7.1990	6.5480	-0.3660
41	40	C13	CT	M	-9.9480	7.6390	7.8690	0.0290
42	41	O6	OS	S	-10.6000	8.8030	8.1160	-0.2780
43	42	C17	CT	3	-12.0780	8.6650	8.1600	-0.0250
44	43	H20	H1	E	-12.5210	9.6180	8.3620	0.0750
45	43	H21	H1	E	-12.4320	8.2940	7.2250	0.0750
46	43	H22	H1	E	-12.3460	7.9790	8.9400	0.0750
47	41	H13	H2	E	-10.3260	6.8980	8.5390	0.1590
48	41	C12	CT	M	-8.4470	7.8290	8.1040	-0.0240
49	48	H12	H1	E	-8.0550	6.8350	8.0310	0.1790
50	48	O5	OS	M	-8.1260	8.3630	9.3840	-0.3250
51	50	C15	CT	M	-8.7590	7.6650	10.4480	0.1360
52	51	H16	H1	E	-8.5020	6.6260	10.4000	0.0260
53	51	H17	H1	E	-9.8190	7.7740	10.3720	0.0260
54	51	C18	CT	M	-8.2770	8.2510	11.7410	0.0700
55	54	H23	H1	E	-8.8430	7.8430	12.5530	0.0450
56	54	H24	H1	E	-8.3880	9.3110	11.7220	0.0450
57	54	O8	OS	M	-6.8910	7.9060	11.8920	-0.2930
58	57	C20	CT	M	-6.4660	8.4580	13.1480	0.0150
59	58	H27	H1	E	-7.0950	8.1100	13.9390	0.0560
60	58	H28	H1	E	-6.5000	9.5280	13.1100	0.0560
61	58	C22	CT	M	-5.0860	8.0010	13.3570	0.1810
62	61	H31	H1	E	-5.0820	6.9340	13.3550	0.0270
63	61	H32	H1	E	-4.7560	8.3600	14.3110	0.0270
64	61	O10	OS	M	-4.1750	8.4500	12.3830	-0.3260
65	64	C24	CA	M	-2.8580	8.1910	12.4970	0.2220
66	65	C23	CA	M	-2.0550	8.6050	11.4340	0.1770
67	66	C25	CA	M	-0.6770	8.3890	11.4740	-0.1690
68	67	H33	HA	E	-0.0630	8.7060	10.6580	0.1330
69	67	C27	CA	M	-0.1020	7.7600	12.5790	-0.2180
70	69	H35	HA	E	0.9550	7.5980	12.6100	0.1620
71	69	C28	CA	M	-0.9030	7.3460	13.6430	-0.2030
72	71	H36	HA	E	-0.4610	6.8660	14.4890	0.1600
73	71	C26	CA	M	-2.2810	7.5610	13.6010	-0.1770
74	73	H34	HA	E	-2.8960	7.2430	14.4170	0.1170

DOXWAOg

1	0	C8	CT	M	-6.1080	2.9310	10.6590	-0.0210
2	1	H9	H1	E	-6.9980	2.6120	10.1590	0.1130
3	1	H10	H1	E	-5.3040	2.2670	10.4120	0.1130
4	1	H11	H1	E	-6.2620	2.9360	11.7100	0.1130
5	1	O2	OS	M	-5.2690	4.1130	10.6660	-0.3960
6	5	C3	C	M	-5.5390	5.1820	9.9150	0.7680
7	6	O1	O	E	-6.5460	5.1820	9.2180	-0.5640
8	6	C1	CT	M	-4.5370	6.2680	10.0790	-0.0150
9	8	N1	N3	3	-5.0880	7.5610	9.5760	-0.2320

10	9	H2	H	E	-4.3900	8.2870	9.6730	0.2910
11	9	H3	H	E	-5.9070	7.8120	10.1110	0.2910
12	9	H4	H	E	-5.3390	7.4680	8.6020	0.2910
13	8	H1	HP	E	-4.2890	6.4050	11.1110	0.1260
14	8	C2	CA	M	-3.2950	5.8630	9.2900	-0.0810
15	14	C4	CA	M	-3.2930	5.7850	7.8970	-0.1210
16	15	H5	HA	E	-4.1650	6.0660	7.3440	0.1480
17	15	C6	CA	M	-2.1520	5.3540	7.2230	-0.1320
18	17	H7	HA	E	-2.1480	5.2980	6.1530	0.1640
19	17	C9	CA	M	-1.0110	4.9920	7.9420	-0.0720
20	19	H12	HA	E	-0.1370	4.6590	7.4250	0.1590
21	19	C7	CA	M	-1.0150	5.0610	9.3350	-0.1320
22	21	H8	HA	E	-0.1410	4.7860	9.8870	0.1640
23	21	C5	CA	M	-2.1560	5.5010	10.0090	-0.1210
24	23	H6	HA	E	-2.1560	5.5530	11.0780	0.1480

FANJAGh

1	0	C1	CA	M	-5.0230	6.5700	9.7810	0.1090
2	1	O1	OS	M	-4.2270	5.6030	9.2290	-0.1870
3	2	C2	CT	M	-4.0580	5.6030	7.8090	0.0300
4	3	H1	H1	E	-3.6090	6.4350	7.5150	0.0640
5	3	H2	H1	E	-4.9360	5.5410	7.3570	0.0640
6	3	C5	CT	M	-3.2130	4.4090	7.4770	0.0300
7	6	H4	H1	E	-2.5030	4.2910	8.1570	0.0640
8	6	H5	H1	E	-2.7860	4.5250	6.5920	0.0640
9	6	O2	OS	M	-4.0780	3.2740	7.4640	-0.1870
10	9	C11	CA	M	-3.5140	2.0230	7.4140	0.1090
11	10	C15	CA	B	-2.1370	1.8040	7.4270	-0.1490
12	11	H12	HA	E	-1.5340	2.5380	7.4620	0.1180
13	11	C20	CA	B	-1.6580	0.5080	7.3870	-0.1820
14	13	C24	CA	B	-2.5280	-0.5600	7.3130	-0.1870
15	14	C19	CA	S	-3.9000	-0.3230	7.3190	-0.1350
16	15	H17	HA	E	-4.4970	-1.0630	7.2870	0.1440
17	14	H22	HA	E	-2.1950	-1.4480	7.2600	0.1460
18	13	H18	HA	E	-0.7210	0.3510	7.4090	0.1550
19	10	C14	CA	M	-4.4180	0.9570	7.3700	0.0160
20	19	C18	CT	M	-5.9120	1.2300	7.3620	-0.0450
21	20	H15	HC	E	-6.0780	2.0930	7.8200	0.0450
22	20	H16	HC	E	-6.2100	1.3300	6.4230	0.0450
23	20	C23	CA	M	-6.7480	0.1650	8.0220	-0.0150
24	23	C27	CA	B	-7.3740	-0.8300	7.2810	-0.2450
25	24	H23	HA	E	-7.2810	-0.8340	6.3360	0.1700
26	24	C31	CA	B	-8.1290	-1.8130	7.8890	-0.1650
27	26	C34	CA	S	-8.2860	-1.7920	9.2610	-0.2450
28	27	H29	HA	E	-8.8130	-2.4640	9.6780	0.1700
29	26	H26	HA	E	-8.5360	-2.4960	7.3690	0.1410
30	23	C26	CA	M	-6.9110	0.1630	9.4200	0.2620
31	30	O4	OH	S	-6.3110	1.1780	10.1060	-0.5100
32	31	H25	HO	E	-5.8970	0.9470	10.8380	0.3490
33	30	C30	CA	M	-7.6910	-0.8120	10.0460	-0.0150
34	33	C35	CT	M	-7.8870	-0.8320	11.5460	-0.0450
35	34	H30	HC	E	-7.7790	0.0900	11.8910	0.0450
36	34	H31	HC	E	-8.8140	-1.1170	11.7390	0.0450
37	34	C38	CA	M	-6.9370	-1.7400	12.2940	0.0160
38	37	C41	CA	B	-6.1420	-2.6930	11.6710	-0.1350
39	38	H37	HA	E	-6.1800	-2.7760	10.7250	0.1440
40	38	C43	CA	B	-5.3010	-3.5200	12.3810	-0.1870
41	40	C44	CA	B	-5.2220	-3.4050	13.7440	-0.1820
42	41	C42	CA	S	-5.9970	-2.4700	14.4150	-0.1490
43	42	H38	HA	E	-5.9390	-2.3920	15.3610	0.1180
44	41	H40	HA	E	-4.6340	-3.9700	14.2320	0.1550
45	40	H39	HA	E	-4.7760	-4.1690	11.9260	0.1460
46	37	C40	CA	M	-6.8550	-1.6570	13.7030	0.1090
47	46	O6	OS	M	-7.6510	-0.6890	14.2550	-0.1870
48	47	C39	CT	M	-7.8200	-0.6900	15.6740	0.0300
49	48	H35	H1	E	-6.9420	-0.6270	16.1260	0.0640
50	48	H36	H1	E	-8.2690	-1.5220	15.9690	0.0640
51	48	C36	CT	M	-8.6650	0.5040	16.0060	0.0300
52	51	H32	H1	E	-9.3750	0.6220	15.3270	0.0640
53	51	H33	H1	E	-9.0920	0.3880	16.8920	0.0640
54	51	O5	OS	M	-7.8000	1.6390	16.0190	-0.1870
55	54	C28	CA	M	-8.3640	2.8900	16.0700	0.1090

56	55	C32	CA	B	-9.7410	3.1090	16.0570	-0.1490
57	56	H27	HA	E	-10.3440	2.3750	16.0220	0.1180
58	56	C37	CA	B	-10.2200	4.4060	16.0970	-0.1820
59	58	C33	CA	B	-9.3500	5.4730	16.1710	-0.1870
60	59	C29	CA	S	-7.9780	5.2370	16.1650	-0.1350
61	60	H24	HA	E	-7.3810	5.9760	16.1970	0.1440
62	59	H28	HA	E	-9.6830	6.3610	16.2240	0.1460
63	58	H34	HA	E	-11.1570	4.5620	16.0740	0.1550
64	55	C25	CA	M	-7.4600	3.9560	16.1130	0.0160
65	64	C22	CT	M	-5.9660	3.6830	16.1210	-0.0450
66	65	H20	HC	E	-5.6680	3.5830	17.0610	0.0450
67	65	H21	HC	E	-5.8000	2.8210	15.6630	0.0450
68	65	C16	CA	M	-5.1300	4.7490	15.4620	-0.0150
69	68	C21	CA	B	-4.5040	5.7430	16.2020	-0.2450
70	69	C17	CA	B	-3.7490	6.7260	15.5940	-0.1650
71	70	C13	CA	S	-3.5920	6.7050	14.2230	-0.2450
72	71	H11	HA	E	-3.0650	7.3770	13.8060	0.1700
73	70	H14	HA	E	-3.3420	7.4090	16.1140	0.1410
74	69	H19	HA	E	-4.5970	5.7470	17.1480	0.1700
75	68	C12	CA	M	-4.9670	4.7500	14.0630	0.2620
76	75	O3	OH	S	-5.5670	3.7350	13.3770	-0.5100
77	76	H13	HO	E	-5.9810	3.9660	12.6460	0.3490
78	75	C9	CA	M	-4.1870	5.7250	13.4370	-0.0150
79	78	C6	CT	M	-3.9910	5.7450	11.9380	-0.0450
80	79	H6	HC	E	-4.0990	4.8230	11.5930	0.0450
81	79	H7	HC	E	-3.0640	6.0300	11.7440	0.0450
82	79	C3	CA	M	-4.9410	6.6530	11.1900	0.0160
83	82	C7	CA	M	-5.7360	7.6060	11.8120	-0.1350
84	83	H8	HA	E	-5.6980	7.6890	12.7590	0.1440
85	83	C10	CA	M	-6.5770	8.4330	11.1030	-0.1870
86	85	H10	HA	E	-7.1020	9.0820	11.5570	0.1460
87	85	C8	CA	M	-6.6560	8.3180	9.7400	-0.1820
88	87	H9	HA	E	-7.2440	8.8830	9.2510	0.1550
89	87	C4	CA	M	-5.8810	7.3830	9.0680	-0.1490
90	89	H3	HA	E	-5.9390	7.3050	8.1230	0.1180

FANJAGg

1	0	N1	NT	M	-6.9520	3.9940	11.1320	-1.0490
2	1	H1	H	E	-6.7170	4.6870	10.4850	0.3760
3	1	H2	H	E	-6.8040	3.2500	10.7080	0.3760
4	1	C1	CT	M	-8.3790	4.0890	11.5090	0.4550
5	4	H3	H1	E	-8.5090	5.0470	11.7660	0.0250
6	4	C3	CT	3	-8.7810	3.3750	12.6170	-0.3150
7	6	H6	HC	E	-8.1900	3.5820	13.3700	0.0760
8	6	H7	HC	E	-8.7350	2.4160	12.4190	0.0760
9	6	H8	HC	E	-9.7020	3.6180	12.8470	0.0760
10	4	C2	CT	M	-9.3420	3.8680	10.3270	-0.1040
11	10	H4	HC	E	-9.2200	2.9450	9.9920	0.0310
12	10	H5	HC	E	-10.2710	3.9440	10.6600	0.0310
13	10	C4	CA	M	-9.1730	4.8050	9.1950	0.0150
14	13	C5	CA	M	-8.3830	4.5180	8.1110	-0.1230
15	14	H9	HA	E	-7.9330	3.6820	8.0720	0.1120
16	14	C7	CA	M	-8.2270	5.4180	7.0770	-0.1720
17	16	H11	HA	E	-7.6730	5.1880	6.3410	0.1450
18	16	C9	CA	M	-8.8420	6.6120	7.0870	-0.1270
19	18	H13	HA	E	-8.7100	7.2040	6.3570	0.1360
20	18	C8	CA	M	-9.6120	6.9810	8.0680	-0.1720
21	20	H12	HA	E	-10.0310	7.8330	8.0470	0.1450
22	20	C6	CA	M	-9.8210	6.1290	9.1470	-0.1230
23	22	H10	HA	E	-10.3870	6.4030	9.8580	0.1120

GUGGUKh

1	0	O6	OH	M	2.0000	-1.2840	-9.3380	-0.5110
2	1	H11	HO	E	3.3720	-0.9840	-9.7390	0.4260
3	1	C13	CA	M	1.2880	-0.2030	-8.9510	0.1550
4	3	C10	CA	M	0.3960	-0.2700	-7.8620	0.0360
5	4	C14	CA	S	-0.3260	0.8610	-7.5000	-0.2780
6	5	H9	HA	E	-0.9250	0.8150	-6.7890	0.1830
7	4	C8	CT	M	0.2430	-1.5350	-7.0430	0.0080
8	7	H5	HC	E	0.2600	-2.3020	-7.6360	-0.0030
9	7	H6	HC	E	-0.6180	-1.5230	-6.5970	-0.0030

10	7	C5	CA	M	1.3420	-1.6850	-6.0010	0.0840
11	10	C6	CA	S	2.5460	-2.3510	-6.3070	0.1180
12	11	O4	OH	S	2.7430	-2.8720	-7.5440	-0.5710
13	12	H7	HO	E	2.2770	-2.3750	-8.3750	0.3680
14	10	C3	CA	M	1.1710	-1.1300	-4.7370	-0.2810
15	14	H2	HA	E	0.3870	-0.6720	-4.5320	0.1980
16	14	C1	CA	M	2.1770	-1.2610	-3.7770	-0.0390
17	16	S1	S6	3	2.0770	-0.4290	-2.2310	1.1610
18	17	O1	O	E	2.5860	-1.3260	-1.2000	-0.7120
19	17	O2	O	E	0.6700	-0.0620	-2.0300	-0.7120
20	17	O3	O	E	2.9030	0.7740	-2.3650	-0.7120
21	16	C2	CA	M	3.3460	-1.9530	-4.0760	-0.2810
22	21	H1	HA	E	4.0000	-2.0470	-3.4230	0.1980
23	21	C4	CA	M	3.5530	-2.5030	-5.3320	0.0840
24	23	C7	CT	M	4.8800	-3.1640	-5.6520	0.0080
25	24	H3	HC	E	4.7740	-3.7730	-6.4010	-0.0030
26	24	H4	HC	E	5.1880	-3.6740	-4.8870	-0.0030
27	24	C9	CA	M	5.8950	-2.0830	-6.0010	0.0360
28	27	C12	CA	S	6.1350	-1.6900	-7.3220	0.1550
29	28	O5	OH	S	5.6240	-2.4890	-8.3100	-0.5110
30	29	H10	HO	E	5.2330	-1.6760	-9.1110	0.4260
31	27	C11	CA	M	6.5130	-1.3730	-4.9650	-0.2780
32	31	H8	HA	E	6.3930	-1.6370	-4.0810	0.1830
33	31	C15	CA	M	7.3030	-0.2740	-5.2610	-0.0280
34	33	S2	S6	3	8.1230	0.6030	-3.9770	1.1620
35	34	O7	O	E	9.5520	0.4270	-4.2050	-0.7200
36	34	O8	O	E	7.7150	-0.0140	-2.7070	-0.7200
37	34	O9	O	E	7.6770	1.9850	-4.0810	-0.7200
38	33	C19	CA	M	7.4760	0.1540	-6.5770	-0.2740
39	38	H12	HA	E	7.9920	0.9080	-6.7530	0.1900
40	38	C16	CA	M	6.8860	-0.5360	-7.6290	0.0110
41	40	C20	CT	M	7.0730	-0.0280	-9.0500	0.0010
42	41	H13	HC	E	7.9590	0.3580	-9.1300	-0.0060
43	41	H14	HC	E	7.0230	-0.7800	-9.6610	-0.0060
44	41	C23	CA	M	6.0390	1.0160	-9.4640	0.0100
45	44	C26	C	S	4.7570	0.6210	-9.8770	0.2450
46	45	O13	O	E	4.4640	-0.7180	-9.9050	-0.7410
47	44	C25	CA	M	6.3520	2.3750	-9.4710	-0.2450
48	47	H18	HA	E	7.2040	2.6550	-9.2300	0.1880
49	47	C28	CA	M	5.3890	3.3200	-9.8390	-0.1210
50	49	S4	S6	3	5.7610	5.0470	-9.9510	1.1650
51	50	O14	O	E	6.8630	5.3150	-9.0330	-0.7210
52	50	O15	O	E	6.1100	5.3300	-11.3370	-0.7210
53	50	O16	O	E	4.5300	5.7360	-9.5470	-0.7210
54	49	C27	CA	M	4.1280	2.9030	-10.2410	-0.2450
55	54	H19	HA	E	3.4920	3.5380	-10.4800	0.1880
56	54	C24	CA	M	3.7990	1.5580	-10.2930	0.0100
57	56	C21	CT	M	2.4340	1.1250	-10.7970	0.0010
58	57	H15	HC	E	2.5110	0.2670	-11.2410	-0.0060
59	57	H16	HC	E	2.1090	1.7690	-11.4440	-0.0060
60	57	C17	CA	M	1.4460	1.0150	-9.6560	0.0110
61	60	C22	CA	M	0.7190	2.1270	-9.2640	-0.2740
62	61	H17	HA	E	0.8220	2.9270	-9.7250	0.1900
63	61	C18	CA	M	-0.1670	2.0630	-8.1860	-0.0280
64	63	S3	S6	M	-1.0570	3.5000	-7.7390	1.1620
65	64	O11	O	E	-1.9030	3.8560	-8.8670	-0.7200
66	64	O12	O	E	-0.0500	4.5290	-7.4670	-0.7200
67	64	O10	O	M	-1.8220	3.1500	-6.5430	-0.7200

GUGGUKg

1	0	N1	N3	M	5.1590	2.6640	-5.6040	-0.3350
2	1	H1	H	E	5.0250	3.1320	-4.7830	0.3540
3	1	H2	H	E	6.1960	2.3450	-5.5760	0.3540
4	1	H3	H	E	4.8300	3.3790	-6.2450	0.3540
5	1	C1	CT	M	4.3160	1.4520	-5.8300	-0.0190
6	5	H4	HP	E	4.4490	0.8310	-5.0990	0.1470
7	5	H5	HP	E	4.6030	1.0160	-6.6480	0.1470
8	5	C2	CT	M	2.8500	1.7780	-5.9380	-0.0190
9	8	H6	HP	E	2.7170	2.4240	-6.6500	0.1470
10	8	H7	HP	E	2.3610	0.9740	-6.1710	0.1470
11	8	N2	N3	M	2.3080	2.3300	-4.6750	-0.3350
12	11	H8	H	E	2.4760	3.0700	-4.4440	0.3540

13	11	H9	H	E	1.2920	2.3170	-4.6580	0.3540
14	11	H10	H	E	2.5490	1.7140	-3.9250	0.3540

HASWUTh

1	0	C12	CT	M	0.0560	-2.5730	0.5520	0.0380
2	1	C13	CT	3	-1.4170	-2.2140	0.3060	0.0380
3	2	N4	N2	S	-1.6360	-1.0920	1.2160	-0.4230
4	3	H8	H	E	-2.2810	-0.6010	1.1480	0.3650
5	2	H12	H1	E	-1.5540	-1.8560	-0.6060	0.1040
6	2	H13	H1	E	-1.9110	-2.9700	0.4560	0.1040
7	1	H10	H1	E	0.0990	-3.4030	1.1480	0.1040
8	1	H11	H1	E	0.5810	-2.8020	-0.2870	0.1040
9	1	N3	N2	M	0.5440	-1.3870	1.2340	-0.4230
10	9	H7	H	E	1.4200	-1.2110	1.4570	0.3650
11	9	C6	CM	M	-0.4680	-0.6240	1.6290	0.3950
12	11	N1	N2	M	-0.3080	0.4750	2.3600	-0.3640
13	12	H4	H	E	0.4070	0.6810	2.5190	0.3160
14	12	C1	CT	M	-1.4040	1.3710	2.7230	0.0320
15	14	H1	H1	E	-1.8960	1.5640	1.9010	0.1120
16	14	C2	CT	M	-2.3760	0.8240	3.7790	-0.0860
17	16	H2	HC	E	-3.2180	0.5300	3.3830	0.0640
18	16	H3	HC	E	-1.9300	0.0880	4.2950	0.0640
19	16	C4	CT	M	-2.6550	1.9980	4.7420	-0.0380
20	19	H5	HC	E	-3.5180	2.4830	4.4010	0.0670
21	19	H6	HC	E	-2.6460	1.6880	5.6440	0.0670
22	19	C5	CA	M	-1.5010	2.9380	4.5190	-0.0390
23	22	C7	CA	S	-1.0380	3.9860	5.3160	-0.0510
24	23	C10	CA	S	-1.6810	4.3660	6.6170	0.0200
25	24	C15	CA	B	-1.0210	4.1800	7.8130	-0.1060
26	25	C18	CA	B	-1.5950	4.5360	9.0080	-0.1730
27	26	C21	CA	B	-2.8470	5.0920	9.0270	-0.0770
28	27	C19	CA	B	-3.5290	5.2710	7.8700	-0.1730
29	28	C16	CA	S	-2.9460	4.9210	6.6450	-0.1060
30	29	H17	HA	E	-3.4240	5.0460	5.8040	0.1380
31	28	H22	HA	E	-4.3430	5.6390	7.8600	0.1650
32	27	H23	HA	E	-3.2620	5.3470	9.9170	0.1560
33	26	H21	HA	E	-1.0900	4.3480	9.8850	0.1650
34	25	H16	HA	E	-0.2660	3.7830	7.8250	0.1380
35	22	C3	CA	M	-0.8350	2.6100	3.3480	0.3010
36	35	N2	NC	M	0.2520	3.2140	2.8850	-0.6290
37	36	C8	CA	M	0.7160	4.1710	3.6860	0.3010
38	37	C9	CA	M	0.1390	4.6090	4.8760	-0.0390
39	38	C14	CT	M	0.9840	5.6960	5.4660	-0.0380
40	39	H14	HC	E	1.0600	5.6740	6.4030	0.0670
41	39	H15	HC	E	0.6170	6.6370	5.1020	0.0670
42	39	C17	CT	M	2.3590	5.4400	4.8210	-0.0860
43	42	H18	HC	E	2.8390	4.7020	5.2930	0.0640
44	42	H19	HC	E	2.9300	6.2220	4.8440	0.0640
45	42	C11	CT	M	2.0200	4.8880	3.4340	0.0320
46	45	H9	H1	E	1.8540	5.6120	2.7390	0.1120
47	45	N5	N2	M	2.9990	3.9590	2.8510	-0.3640
48	47	H20	H	E	2.7240	3.1020	2.8190	0.3160
49	47	C20	CM	M	4.1970	4.3470	2.4430	0.3950
50	49	N6	N2	M	5.1040	3.4930	1.9770	-0.4230
51	50	H24	H	E	4.9650	2.5450	2.1360	0.3650
52	50	C22	CT	M	6.3570	4.1880	1.7250	0.0380
53	52	H26	H1	E	7.0670	3.9420	2.3600	0.1040
54	52	H27	H1	E	6.6910	3.9860	0.7330	0.1040
55	52	C23	CT	M	5.9560	5.6590	1.7860	0.0380
56	55	H28	H1	E	5.8140	6.0810	0.8290	0.1040
57	55	H29	H1	E	6.4220	6.2130	2.2640	0.1040
58	55	N7	N2	M	4.6430	5.5780	2.4550	-0.4230
59	58	H25	H	E	4.1010	6.2570	2.4040	0.3650

HASWUTg

1	0	O1	O	M	2.3900	1.1910	3.0160	-0.8720
2	1	P1	P	M	3.5300	0.2290	2.8290	1.2900
3	2	O2	O	E	3.1330	-1.0410	2.0930	-0.8720
4	2	O3	O	E	4.7750	0.8730	2.2740	-0.8720
5	2	O4	OS	M	3.9540	-0.3720	4.2910	-0.6510
6	5	C1	CA	M	4.5080	0.3640	5.3090	0.5690

7	6	C2	CA	M	4.3160	1.7300	5.4850	-0.2110
8	7	H1	HA	E	3.8420	2.2100	4.8250	0.1020
9	7	C4	CA	M	4.8820	2.3690	6.5530	-0.2050
10	9	H3	HA	E	4.6940	3.2970	6.6450	0.1130
11	9	C6	CA	M	5.6230	1.6780	7.4900	-0.2950
12	11	H5	HA	E	5.9770	2.1920	8.1950	0.1030
13	11	C5	CA	M	5.8000	0.3280	7.3120	-0.2050
14	13	H4	HA	E	6.3040	-0.1680	7.8130	0.1130
15	13	C3	CA	M	5.2660	-0.3380	6.2400	-0.2110
16	15	H2	HA	E	5.3810	-1.2550	6.0590	0.1020

JEJWOKh

1	0	C9	CT	M	-2.7850	7.1600	3.7100	0.0030
2	1	H13	H1	E	-1.8000	6.8890	4.0900	0.0550
3	1	H64	H1	E	-3.5190	6.4310	4.0530	0.0550
4	1	H82	H1	E	-2.7630	7.1700	2.6200	0.0550
5	1	O5	OS	M	-3.1540	8.5070	4.2100	-0.3000
6	5	C6	CT	M	-2.6270	8.8140	5.4640	-0.0170
7	6	H6	H1	E	-3.2280	9.5390	5.9180	0.0860
8	6	H7	H1	E	-2.7150	8.0710	6.0240	0.0860
9	6	C5	CT	M	-1.1440	9.3420	5.3750	0.0200
10	9	O4	OS	E	-1.2760	10.6230	4.7420	-0.3150
11	9	H5	H1	E	-0.4070	8.7580	4.7930	0.1480
12	9	C4	CT	M	-0.5380	9.4840	6.7770	-0.0070
13	12	O3	OS	E	-0.1610	8.1940	7.2540	-0.2360
14	12	H4	H1	E	-1.1440	9.9170	7.4410	0.1440
15	12	C3	CT	M	0.7140	10.3550	6.7520	-0.0350
16	15	O2	OS	S	0.9190	10.7740	8.1380	-0.3450
17	16	C8	CT	3	2.2710	11.0530	8.5330	-0.0150
18	17	H12	H1	E	2.8850	11.2000	7.6450	0.0650
19	17	H63	H1	E	2.6620	10.2150	9.1100	0.0650
20	17	H81	H1	E	2.2930	11.9560	9.1430	0.0650
21	15	H3	H1	E	1.5830	9.8230	6.4740	0.1660
22	15	C2	CT	M	0.6010	11.5770	5.8530	0.0310
23	22	O1	OS	S	1.8770	12.1560	5.7410	-0.2340
24	23	C7	CT	3	1.8640	13.5650	5.4550	-0.1270
25	24	H11	H1	E	1.6670	14.1200	6.3720	0.0890
26	24	H62	H1	E	1.0830	13.7810	4.7260	0.0890
27	24	H80	H1	E	2.8320	13.8630	5.0520	0.0890
28	22	H2	H1	E	0.1220	12.2610	6.2760	0.1470
29	22	C1	CT	M	-0.0100	11.2590	4.4900	0.0060
30	29	H1	H2	E	-0.1320	12.1900	3.8390	0.1690
31	29	O8	OS	M	0.8190	10.4140	3.7520	-0.2360
32	31	C13	CT	M	0.9080	10.6420	2.3220	-0.0070
33	32	C14	CT	3	-0.0810	9.7330	1.5490	0.0200
34	33	O9	OS	E	0.2560	9.6760	0.1710	-0.3150
35	33	C15	CT	3	-1.5290	10.2890	1.5950	-0.0170
36	35	O10	OS	S	-2.3730	9.3990	0.9330	-0.3000
37	36	C18	CT	3	-3.7110	9.8460	0.9520	0.0030
38	37	H16	H1	E	-4.3390	9.0920	1.4260	0.0550
39	37	H67	H1	E	-3.7760	10.7770	1.5140	0.0550
40	37	H85	H1	E	-4.0530	10.0130	-0.0690	0.0550
41	35	H33	H1	E	-1.8280	10.2960	2.5680	0.0860
42	35	H34	H1	E	-1.6450	11.1950	1.1120	0.0860
43	33	H32	H1	E	-0.0710	8.8050	2.0120	0.1480
44	32	H31	H1	E	0.6940	11.6450	2.1320	0.1440
45	32	C12	CT	M	2.3590	10.3550	1.9730	-0.0350
46	45	O7	OS	S	3.1520	11.4010	2.4640	-0.3450
47	46	C17	CT	3	4.1690	10.9540	3.4240	-0.0150
48	47	H15	H1	E	4.4860	9.9420	3.1730	0.0650
49	47	H66	H1	E	3.7500	10.9650	4.4300	0.0650
50	47	H84	H1	E	5.0280	11.6240	3.3810	0.0650
51	45	H30	H1	E	2.7110	9.5620	2.4490	0.1660
52	45	C11	CT	M	2.5230	10.2840	0.4510	0.0310
53	52	O6	OS	S	3.8570	9.9980	0.1030	-0.2340
54	53	C16	CT	3	4.4320	10.8810	-0.7750	-0.1270
55	54	H14	H1	E	4.4950	11.8660	-0.3120	0.0890
56	54	H65	H1	E	5.4340	10.5360	-1.0320	0.0890
57	54	H83	H1	E	3.8260	10.9420	-1.6790	0.0890
58	52	H9	H1	E	2.3980	11.1720	0.0130	0.1470
59	52	C10	CT	M	1.5680	9.2500	-0.1170	0.0060
60	59	H8	H2	E	1.6690	9.1360	-1.1120	0.1690

61	59	O13	OS	M	1.8230	7.9810	0.4830	-0.2360
62	61	C22	CT	M	1.9260	6.8210	-0.3380	-0.0070
63	62	C23	CT	3	0.6400	6.0210	-0.3470	0.0200
64	63	O14	OS	E	0.8340	4.8020	-1.1080	-0.3150
65	63	C24	CT	3	-0.4970	6.7600	-1.1540	-0.0170
66	65	O15	OS	S	-1.7470	6.0120	-0.9070	-0.3000
67	66	C27	CT	3	-2.8770	6.6060	-1.0750	0.0030
68	67	H19	H1	E	-2.7620	7.6710	-0.8750	0.0550
69	67	H70	H1	E	-3.6130	6.1860	-0.3900	0.0550
70	67	H88	H1	E	-3.2140	6.4630	-2.1020	0.0550
71	65	H40	H1	E	-0.6050	7.7160	-0.7680	0.0860
72	65	H41	H1	E	-0.2130	6.8170	-2.3430	0.0860
73	63	H39	H1	E	0.4270	5.8230	0.7020	0.1480
74	62	H38	H1	E	2.1730	7.1010	-1.2840	0.1440
75	62	C21	CT	M	3.0580	5.9620	0.2360	-0.0350
76	75	O12	OS	S	4.3230	6.6510	0.1270	-0.3450
77	76	C26	CT	3	4.8920	6.9940	1.4390	-0.0150
78	77	H18	H1	E	5.1620	6.0800	1.9690	0.0650
79	77	H69	H1	E	4.1560	7.5480	2.0210	0.0650
80	77	H87	H1	E	5.7810	7.6080	1.2990	0.0650
81	75	H37	H1	E	2.8590	5.8230	1.3370	0.1660
82	75	C20	CT	M	3.1890	4.6490	-0.4830	0.0310
83	82	O11	OS	S	4.1210	3.8010	0.1500	-0.2340
84	83	C25	CT	3	5.3080	3.5550	-0.5200	-0.1270
85	84	H17	H1	E	6.1020	3.3740	0.2040	0.0890
86	84	H68	H1	E	5.5660	4.4190	-1.1330	0.0890
87	84	H86	H1	E	5.1910	2.6780	-1.1570	0.0890
88	82	H36	H1	E	3.6710	4.8050	-1.4030	0.1470
89	82	C19	CT	M	1.7720	3.9010	-0.5440	0.0060
90	89	H35	H2	E	1.6580	2.9820	-1.1920	0.1690
91	89	O18	OS	M	1.3920	3.5050	0.7350	-0.2360
92	91	C31	CT	M	0.7490	2.2110	0.8530	-0.0070
93	92	C32	CT	3	-0.6510	2.4280	1.4590	0.0200
94	93	O19	OS	E	-1.1760	1.1760	1.9040	-0.3150
95	93	C33	CT	3	-1.6970	2.9870	0.4810	-0.0170
96	95	O20	OS	S	-1.7350	2.1400	-0.6470	-0.3000
97	96	C36	CT	3	-2.8090	2.6180	-1.5560	0.0030
98	97	H22	H1	E	-2.4280	3.4310	-2.1740	0.0550
99	97	H73	H1	E	-3.1370	1.7980	-2.1950	0.0550
100	97	H91	H1	E	-3.6520	2.9770	-0.9650	0.0550
101	95	H47	H1	E	-1.5140	3.9290	0.2120	0.0860
102	95	H48	H1	E	-2.8740	3.0770	0.9140	0.0860
103	93	H46	H1	E	-0.5490	3.1010	2.3300	0.1480
104	92	H45	H1	E	0.6970	1.7750	-0.1190	0.1440
105	92	C30	CT	M	1.6550	1.3400	1.7580	-0.0350
106	105	O17	OS	S	2.7900	0.9890	0.9840	-0.3450
107	106	C35	CT	3	4.0700	1.3370	1.7570	-0.0150
108	107	H21	H1	E	4.8330	1.6830	1.0590	0.0650
109	107	H72	H1	E	4.4310	0.4520	2.2800	0.0650
110	107	H90	H1	E	3.8560	2.1240	2.4800	0.0650
111	105	H44	H1	E	2.0950	1.8460	2.5290	0.1660
112	105	C29	CT	M	0.9300	0.1230	2.2610	0.0310
113	112	O16	OS	S	1.7340	-0.5750	3.1630	-0.2340
114	113	C34	CT	3	1.4340	-1.9380	3.3140	-0.1270
115	114	H20	H1	E	0.4740	-2.1520	2.8440	0.0890
116	114	H71	H1	E	1.3810	-2.1840	4.3750	0.0890
117	114	H89	H1	E	2.2120	-2.5370	2.8400	0.0890
118	112	H43	H1	E	0.7920	-0.5680	1.5760	0.1470
119	112	C28	CT	M	-0.3860	0.5250	2.9030	0.0060
120	119	H42	H2	E	-0.8200	-0.3790	3.2300	0.1690
121	119	O23	OS	M	-0.1610	1.4080	3.9850	-0.2360
122	121	C40	CT	M	-0.9470	1.3020	5.1650	-0.0070
123	122	C41	CT	3	-1.8390	2.5040	5.3040	0.0200
124	123	O24	OS	E	-2.4280	2.5490	6.5990	-0.3150
125	123	C42	CT	3	-3.0920	2.3670	4.3360	-0.0170
126	125	O25	OS	S	-3.7130	3.6210	4.2940	-0.3000
127	126	C45	CT	3	-4.9120	3.5690	3.4110	0.0030
128	127	H27	H1	E	-5.2680	2.5410	3.3410	0.0550
129	127	H76	H1	E	-4.6480	3.9310	2.4170	0.0550
130	127	H94	H1	E	-5.6980	4.1970	3.8290	0.0550
131	125	H24	H1	E	-3.8250	1.6330	4.6210	0.0860
132	125	H53	H1	E	-2.8320	2.2250	3.3630	0.0860
133	123	H52	H1	E	-1.2650	3.3140	5.1370	0.1480

134	122	H51	H1	E	-1.5510	0.4500	5.1630	0.1440
135	122	C39	CT	M	0.0860	1.2280	6.3250	-0.0350
136	135	O22	OS	S	0.7060	-0.0470	6.2870	-0.3450
137	136	C44	CT	3	2.1870	0.0070	6.3750	-0.0150
138	137	H26	H1	E	2.5680	0.7210	5.6450	0.0650
139	137	H75	H1	E	2.6000	-0.9800	6.1670	0.0650
140	137	H93	H1	E	2.4810	0.3200	7.3770	0.0650
141	135	H50	H1	E	0.9550	1.9650	6.1700	0.1660
142	135	C38	CT	M	-0.6280	1.4130	7.6840	0.0310
143	142	O21	OS	S	0.3540	1.4890	8.6880	-0.2340
144	143	C43	CT	3	-0.0200	1.0110	9.9320	-0.1270
145	144	H25	H1	E	0.0530	1.8130	10.6670	0.0890
146	144	H74	H1	E	0.6390	0.1920	10.2200	0.0890
147	144	H92	H1	E	-1.0480	0.6520	9.8880	0.0890
148	142	H23	H1	E	-1.1140	0.5680	7.9570	0.1470
149	142	C37	CT	M	-1.5060	2.6750	7.6780	0.0060
150	149	H49	H2	E	-2.0630	2.7690	8.6450	0.1690
151	149	O28	OS	M	-0.6810	3.8230	7.4510	-0.2360
152	151	C49	CT	M	-0.8700	4.9590	8.3180	-0.0070
153	152	H57	H1	E	-1.2370	4.6860	9.2540	0.1440
154	152	C48	CT	M	0.5240	5.5670	8.5400	-0.0350
155	154	O27	OS	S	1.2770	4.7010	9.3590	-0.3450
156	155	C53	CT	3	2.4120	4.0780	8.8100	-0.0150
157	156	H29	H1	E	2.6660	4.5540	7.8630	0.0650
158	156	H78	H1	E	2.2020	3.0220	8.6410	0.0650
159	156	H96	H1	E	3.2490	4.1750	9.5010	0.0650
160	154	H56	H1	E	1.1160	5.6570	7.6790	0.1660
161	154	C47	CT	M	0.4150	6.9440	9.2230	0.0310
162	161	O26	OS	S	1.6880	7.5340	9.2360	-0.2340
163	162	C52	CT	3	2.1710	7.8790	10.4860	-0.1270
164	163	H28	H1	E	2.4400	6.9760	11.0340	0.0890
165	163	H77	H1	E	1.4030	8.4230	11.0360	0.0890
166	163	H95	H1	E	3.0520	8.5110	10.3730	0.0890
167	161	H55	H1	E	0.1590	6.9820	10.2870	0.1470
168	161	C46	CT	M	-0.6220	7.8440	8.5470	0.0060
169	168	H54	H2	E	-0.7410	8.7580	9.1490	0.1690
170	168	O29	OS	M	-1.8610	7.1790	8.4490	-0.3150
171	170	C50	CT	M	-1.8420	5.9390	7.6470	0.0200
172	171	H58	H1	E	-1.4590	6.0360	6.6330	0.1480
173	171	C51	CT	M	-3.2940	5.5170	7.5390	-0.0170
174	173	H59	H1	E	-4.0180	6.4140	7.3480	0.0860
175	173	H60	H1	E	-3.3910	4.8050	6.8450	0.0860
176	173	O30	OS	M	-3.7270	5.0300	8.7840	-0.3000
177	176	C54	CT	M	-5.1550	5.0790	8.8900	0.0030
178	177	H10	H1	E	-5.4850	4.4000	9.6770	0.0550
179	177	H61	H1	E	-5.4670	6.0950	9.1310	0.0550
180	177	H79	H1	E	-5.6000	4.7780	7.9410	0.0550

JEJWOKg

1	0	C7	CT	M	5.4230	7.7540	5.5710	-0.2910
2	1	H7	HC	E	5.6320	8.7720	5.8990	0.0740
3	1	H8	HC	E	5.1230	7.7630	4.5230	0.0740
4	1	H9	HC	E	6.3190	7.1450	5.6870	0.0740
5	1	C4	CT	M	4.3430	7.1910	6.3830	0.3160
6	5	O1	OH	S	3.6020	8.1970	7.1710	-0.6770
7	6	H10	HO	E	2.8800	7.6950	7.8150	0.4260
8	5	H3	H1	E	4.9860	6.7460	7.0830	0.0780
9	5	C1	CA	M	3.3200	6.3950	5.5180	-0.0280
10	9	C2	CA	M	3.4940	5.0860	5.3540	-0.1430
11	10	H1	HA	E	4.6570	4.5920	5.6270	0.1330
12	10	C5	CA	M	2.6960	4.2960	4.6260	-0.1530
13	12	H4	HA	E	2.7310	3.1240	4.6210	0.1350
14	12	C8	CA	M	1.6480	4.9040	3.9850	-0.1190
15	14	H6	HA	E	1.0470	4.4260	3.4290	0.1300
16	14	C6	CA	M	1.4040	6.2530	4.0550	-0.1530
17	16	H5	HA	E	0.5600	6.6980	3.3500	0.1350
18	16	C3	CA	M	2.3020	7.0150	4.8660	-0.1430
19	18	H2	HA	E	2.1840	8.0240	4.8990	0.1330

KAXPOOh

1	0	C16	CA	M	6.8240	-8.8540	2.6700	-0.1060

2	1	C11	CA	B	6.6180	-8.4200	1.3850	-0.2100
3	2	C6	CA	S	5.3550	-8.0740	0.9290	-0.0710
4	3	H4	HA	E	5.2300	-7.7880	0.0200	0.1230
5	2	H9	HA	E	7.3700	-8.3540	0.7820	0.1470
6	1	H12	HA	E	7.7070	-9.1000	2.9610	0.1360
7	1	C10	CA	M	5.7610	-8.9200	3.5240	-0.2100
8	7	H8	HA	E	5.9040	-9.2320	4.4140	0.1470
9	7	C5	CA	M	4.4920	-8.5590	3.0820	-0.0710
10	9	H3	HA	E	3.7530	-8.5960	3.7000	0.1230
11	9	C2	CA	M	4.2690	-8.1420	1.7990	0.0500
12	11	C1	CT	M	2.8220	-7.9320	1.3250	0.0390
13	12	O1	OH	S	2.3500	-9.2530	1.0290	-0.5660
14	13	H1	HO	E	2.1180	-9.2390	0.1310	0.3960
15	12	C3	CA	S	1.9520	-7.3000	2.4290	0.0500
16	15	C7	CA	B	0.7410	-7.8500	2.7700	-0.0710
17	16	C12	CA	B	-0.0110	-7.3170	3.8090	-0.2100
18	17	C17	CA	B	0.4060	-6.2230	4.5020	-0.1060
19	18	C13	CA	B	1.6220	-5.6600	4.1460	-0.2100
20	19	C8	CA	S	2.3700	-6.1780	3.1190	-0.0710
21	20	H6	HA	E	3.1930	-5.7520	2.8660	0.1230
22	19	H11	HA	E	1.9570	-4.9100	4.6370	0.1470
23	18	H13	HA	E	-0.1310	-5.8450	5.2020	0.1360
24	17	H10	HA	E	-0.8390	-7.7400	4.0450	0.1470
25	16	H5	HA	E	0.4150	-8.6160	2.2880	0.1230
26	12	C4	CT	M	2.7460	-7.0900	0.0180	0.1150
27	26	H2	H1	E	3.2740	-7.5120	-0.6690	0.0890
28	26	O2	OS	M	3.2840	-5.8040	0.3030	-0.4610
29	28	C14	CT	M	2.6900	-4.8550	-0.5520	0.5530
30	29	C18	CT	3	3.5160	-4.5640	-1.8040	0.0110
31	30	C22	CT	3	4.2520	-3.3590	-1.5090	-0.0560
32	31	C23	CT	3	3.1730	-2.4840	-0.7140	-0.0560
33	32	C19	CT	B	2.5260	-3.5060	0.1420	0.0110
34	33	H16	HC	E	1.5930	-3.3080	0.2490	0.0010
35	33	H17	HC	E	2.9520	-3.5270	1.0010	0.0010
36	32	H21	HC	E	2.5500	-2.0870	-1.3120	0.0190
37	32	H22	HC	E	3.5930	-1.8120	-0.1970	0.0190
38	31	H19	HC	E	4.9970	-3.5060	-0.9770	0.0190
39	31	H20	HC	E	4.4980	-2.8770	-2.3240	0.0190
40	30	H14	HC	E	4.1090	-5.2830	-1.9860	0.0010
41	30	H15	HC	E	2.9440	-4.4300	-2.5520	0.0010
42	29	O3	OS	M	1.3990	-5.4030	-0.8990	-0.4610
43	42	C9	CT	M	1.3390	-6.7760	-0.5210	0.1150
44	43	H7	H1	E	0.7110	-6.8700	0.1970	0.0890
45	43	C15	CT	M	0.8510	-7.6400	-1.7140	0.0390
46	45	O4	OH	S	0.9310	-9.0140	-1.2720	-0.5660
47	46	H18	HO	E	0.1770	-9.2570	-0.9310	0.3960
48	45	C21	CA	S	-0.6270	-7.3440	-2.0920	0.0500
49	48	C26	CA	B	-1.4500	-6.5170	-1.3550	-0.0710
50	49	C30	CA	B	-2.7750	-6.3430	-1.7020	-0.2100
51	50	C33	CA	B	-3.2930	-6.9720	-2.8080	-0.1060
52	51	C31	CA	B	-2.4880	-7.7930	-3.5510	-0.2100
53	52	C27	CA	S	-1.1620	-7.9980	-3.1830	-0.0710
54	53	H26	HA	E	-0.6120	-8.6080	-3.6870	0.1230
55	52	H30	HA	E	-2.8340	-8.2310	-4.3400	0.1470
56	51	H32	HA	E	-4.2140	-6.8360	-3.0590	0.1360
57	50	H29	HA	E	-3.3370	-5.7830	-1.1710	0.1470
58	49	H25	HA	E	-1.0960	-6.0630	-0.5880	0.1230
59	45	C20	CA	M	1.7580	-7.4780	-2.9160	0.0500
60	59	C24	CA	M	1.5820	-6.4300	-3.8110	-0.0710
61	60	H23	HA	E	0.8540	-5.8250	-3.6850	0.1230
62	60	C28	CA	M	2.4320	-6.2450	-4.8780	-0.2100
63	62	H27	HA	E	2.2940	-5.5150	-5.4850	0.1470
64	62	C32	CA	M	3.4730	-7.1060	-5.0810	-0.1060
65	64	H31	HA	E	4.0670	-6.9700	-5.8270	0.1360
66	64	C29	CA	M	3.6740	-8.1680	-4.2260	-0.2100
67	66	H28	HA	E	4.4170	-8.7730	-4.3660	0.1470
68	66	C25	CA	M	2.8130	-8.3680	-3.1520	-0.0710
69	68	H24	HA	E	2.9410	-9.1250	-2.5750	0.1230

KAXPOOg

1	0	C9	CT	M	-3.0840	-0.8450	-2.9740	-0.0590
2	1	H10	HC	E	-3.9850	-0.6660	-2.7740	0.0420

3	1	H11	HC	E	-2.7720	-0.2980	-3.6960	0.0420
4	1	H12	HC	E	-2.9680	-1.7760	-3.2120	0.0420
5	1	C5	CM	M	-2.2400	-0.5710	-1.7650	-0.0840
6	5	H4	HA	E	-2.2340	0.3260	-1.4150	0.1200
7	5	C2	CM	M	-1.4920	-1.5020	-1.1420	-0.1730
8	7	C6	CT	3	-1.3740	-2.9040	-1.5830	-0.0690
9	8	H5	HC	E	-2.2490	-3.3180	-1.5740	0.0400
10	8	H6	HC	E	-1.0230	-2.9390	-2.4750	0.0400
11	8	H7	HC	E	-0.7950	-3.3880	-0.9900	0.0400
12	7	C1	C	M	-0.6840	-1.0370	0.0230	0.5880
13	12	O1	O	E	0.0000	0.0000	0.0000	-0.5790
14	12	N1	N	M	-0.6680	-1.7930	1.1440	-0.1170
15	14	C4	CT	3	0.2800	-1.4600	2.2210	-0.3200
16	15	H1	H1	E	0.1650	-2.0760	2.9490	0.1280
17	15	H2	H1	E	0.1160	-0.5670	2.5290	0.1280
18	15	H3	H1	E	1.1780	-1.5250	1.8880	0.1280
19	14	C3	CA	M	-1.6780	-2.7810	1.4300	0.0440
20	19	C7	CA	M	-1.2900	-4.0690	1.7900	-0.0880
21	20	H8	HA	E	-0.3490	-4.3030	1.8480	0.1190
22	20	C10	CA	M	-2.2550	-5.0130	2.0490	-0.1780
23	22	H13	HA	E	-1.9860	-5.9050	2.3160	0.1460
24	22	C12	CA	M	-3.5850	-4.7130	1.9540	-0.1110
25	24	H15	HA	E	-4.2480	-5.4000	2.1050	0.1340
26	24	C11	CA	M	-3.9670	-3.4450	1.6310	-0.1780
27	26	H14	HA	E	-4.9050	-3.2210	1.5880	0.1460
28	26	C8	CA	M	-3.0050	-2.4520	1.3660	-0.0880
29	28	H9	HA	E	-3.2750	-1.5650	1.1400	0.1190

LAYMAZh

1	0	N1	NC	M	1.0220	-2.0460	6.1290	-0.5700
2	1	C1	CA	M	2.2410	-2.5990	6.2620	0.3450
3	2	C3	C	M	2.8790	-3.2340	5.0760	0.6910
4	3	O1	O	E	3.8200	-3.9540	5.1450	-0.5890
5	3	O2	OS	M	2.2900	-2.8950	3.9270	-0.3980
6	5	C8	CT	M	2.9600	-3.3140	2.6850	0.2480
7	6	C10	CT	3	3.9130	-2.2340	2.2430	-0.1710
8	7	H6	HC	E	3.5230	-1.3930	1.9920	0.0580
9	7	H7	HC	E	4.4230	-2.5820	1.5080	0.0580
10	7	H8	HC	E	4.4880	-2.1050	2.9990	0.0580
11	6	H4	H1	E	3.5110	-4.0940	2.7890	0.0700
12	6	C11	CT	M	1.8810	-3.6640	1.7050	-0.0580
13	12	H9	H1	E	1.3320	-4.3580	2.0760	0.0730
14	12	H10	H1	E	2.2850	-3.9730	0.8910	0.0730
15	12	O5	OS	M	1.0760	-2.5330	1.4260	-0.3080
16	15	C14	CT	M	0.0480	-2.8830	0.4940	0.0050
17	16	H16	H1	E	0.4490	-3.1120	-0.3480	0.0650
18	16	H17	H1	E	-0.4440	-3.6350	0.8310	0.0650
19	16	C16	CT	M	-0.8740	-1.7430	0.3020	0.1960
20	19	H20	H1	E	-1.5380	-1.9760	-0.3510	0.0010
21	19	H21	H1	E	-0.3790	-0.9790	-0.0040	0.0010
22	19	O7	OS	M	-1.5070	-1.4360	1.5230	-0.2990
23	22	C17	CT	M	-2.6640	-0.6150	1.4130	0.1960
24	23	H22	H1	E	-2.7500	-0.3220	0.5040	0.0010
25	23	H23	H1	E	-3.4350	-1.1320	1.6580	0.0010
26	23	C15	CT	M	-2.5880	0.5350	2.2640	0.0050
27	26	H18	H1	E	-1.9240	1.1270	1.9020	0.0650
28	26	H19	H1	E	-3.4440	0.9680	2.2530	0.0650
29	26	O6	OS	M	-2.2400	0.2680	3.6610	-0.3080
30	29	C12	CT	M	-3.1410	-0.5480	4.3560	-0.0580
31	30	H11	H1	E	-4.0330	-0.2220	4.2160	0.0730
32	30	H12	H1	E	-3.0720	-1.4460	4.0250	0.0730
33	30	C9	CT	M	-2.8390	-0.5410	5.8390	0.2480
34	33	C13	CT	3	-3.8350	-1.3390	6.6380	-0.1710
35	34	H13	HC	E	-4.7000	-0.9430	6.5020	0.0580
36	34	H14	HC	E	-3.6530	-1.3950	7.5770	0.0580
37	34	H15	HC	E	-3.8240	-2.2200	6.2560	0.0580
38	33	H5	H1	E	-2.8940	0.3530	6.1820	0.0700
39	33	O3	OS	M	-1.4980	-1.1060	5.9780	-0.3980
40	39	C5	C	M	-0.8680	-0.8870	7.1480	0.6910
41	40	O4	O	E	-1.3600	-0.2740	8.0680	-0.5890
42	40	C2	CA	M	0.4940	-1.4910	7.2250	0.3450
43	42	C6	CA	M	1.1680	-1.4030	8.4360	-0.2750

44	43	H2	HA	E	0.7640	-0.9600	9.1870	0.1630
45	43	C7	CA	M	2.4100	-1.9540	8.5480	-0.0160
46	45	H3	HA	E	2.8980	-1.8960	9.3700	0.1450
47	45	C4	CA	M	2.9450	-2.5920	7.4620	-0.2750
48	47	H1	HA	E	3.7970	-3.0300	7.5270	0.1630

LAYMAZg

1	0	O1	OH	M	0.8850	0.9350	1.3410	-0.6640
2	1	H12	HO	E	1.6310	1.3480	0.6730	0.4790
3	1	C7	CT	M	1.0600	1.5520	2.5920	0.0640
4	3	H9	H1	E	1.7610	2.2060	2.5270	0.0970
5	3	H10	H1	E	0.2420	1.9850	2.8500	0.0970
6	3	C4	CT	M	1.4320	0.5250	3.6450	0.0350
7	6	N1	N3	3	0.4510	-0.6010	3.5900	-0.3380
8	7	H6	H	E	0.6720	-1.3130	4.0500	0.3200
9	7	H7	H	E	-0.4340	-0.4200	3.6810	0.3200
10	7	H8	H	E	0.5800	-0.8700	2.6380	0.3200
11	6	H3	HP	E	2.3150	0.2060	3.4450	0.1660
12	6	C1	CA	M	1.5080	1.1060	5.0390	-0.0270
13	12	C2	CA	M	2.6670	0.9810	5.7680	-0.1610
14	13	H1	HA	E	3.4260	0.5360	5.3820	0.1450
15	13	C5	CA	M	2.7470	1.4880	7.0540	-0.1180
16	15	H4	HA	E	3.5560	1.3850	7.5590	0.1610
17	15	C8	CA	M	1.6730	2.1370	7.6060	-0.0790
18	17	H11	HA	E	1.7240	2.4830	8.5010	0.1540
19	17	C6	CA	M	0.5130	2.2890	6.8640	-0.1180
20	19	H5	HA	E	-0.1320	2.7610	7.2380	0.1610
21	19	C3	CA	M	0.4340	1.7710	5.5990	-0.1610
22	21	H2	HA	E	-0.3780	1.8690	5.0980	0.1450

NOYNAQh

1	0	N1	NA	M	12.3330	1.7670	9.3890	-0.1430
2	1	H1	H	E	12.1670	2.6890	9.5950	0.2690
3	1	C1	CC	M	13.4250	1.0440	9.8340	-0.0490
4	3	C3	C*	M	13.2960	-0.2150	9.3130	-0.2170
5	4	H2	HA	E	13.9160	-0.9360	9.4430	0.1480
6	4	C5	C*	M	12.1140	-0.2670	8.5500	-0.2170
7	6	H3	HA	E	11.7750	-1.0310	8.0780	0.1480
8	6	C2	CC	M	11.5240	0.9820	8.5950	-0.0490
9	8	C6	CT	M	10.3580	1.5750	7.8530	0.0170
10	9	C11	CT	3	10.6350	2.3640	6.6260	0.0110
11	10	C17	CT	3	11.6530	1.5480	5.6020	-0.0430
12	11	H22	HC	E	11.9720	2.0500	4.8470	0.0150
13	11	H23	HC	E	11.0860	0.8450	5.2800	0.0150
14	11	H24	HC	E	12.4110	1.1600	6.0480	0.0150
15	10	H8	HC	E	10.1250	2.7520	6.1800	0.0170
16	10	H9	HC	E	11.4500	3.0670	6.9480	0.0170
17	9	C12	CT	3	9.4060	0.4310	7.4090	0.0110
18	17	C18	CT	3	8.1840	0.9010	6.6200	-0.0430
19	18	H25	HC	E	7.5970	0.1890	6.3530	0.0150
20	18	H26	HC	E	8.4870	1.3600	5.8330	0.0150
21	18	H27	HC	E	7.6890	1.5150	7.1700	0.0150
22	17	H10	HC	E	9.9010	-0.1840	6.8590	0.0170
23	17	H11	HC	E	9.1020	-0.0280	8.1960	0.0170
24	9	C10	CC	M	9.6170	2.5280	8.7670	-0.0490
25	24	C16	C*	B	9.2390	3.8430	8.6160	-0.2170
26	25	C22	C*	S	8.5210	4.2260	9.7660	-0.2170
27	26	H29	HA	E	8.1400	5.0910	9.9360	0.1480
28	25	H21	HA	E	9.4280	4.3960	7.8550	0.1480
29	24	N3	NA	M	9.1340	2.1170	9.9850	-0.1430
30	29	H20	H	E	9.2440	1.2350	10.3480	0.2690
31	29	C21	CC	M	8.4670	3.1440	10.6180	-0.0490
32	31	C24	CT	M	7.7660	2.9370	11.9330	0.0170
33	32	C29	CT	3	7.3720	4.3320	12.4980	0.0110
34	33	C35	CT	3	6.5860	4.2870	13.7980	-0.0430
35	34	H47	HC	E	6.3480	5.1540	14.1320	0.0150
36	34	H48	HC	E	7.1300	3.8400	14.4510	0.0150
37	34	H49	HC	E	5.7840	3.7770	13.6550	0.0150
38	33	H34	HC	E	6.8280	4.7790	11.8440	0.0170
39	33	H35	HC	E	8.1730	4.8420	12.6410	0.0170
40	32	C30	CT	3	6.5050	2.0460	11.7170	0.0110

41	40	C36	CT	3	5.5180	2.5790	10.6840	-0.0430
42	41	H50	HC	E	4.7440	2.0240	10.5570	0.0150
43	41	H51	HC	E	5.2280	3.4490	10.9720	0.0150
44	41	H52	HC	E	5.9780	2.6630	9.8460	0.0150
45	40	H36	HC	E	6.7940	1.1760	11.4290	0.0170
46	40	H37	HC	E	6.0450	1.9620	12.5550	0.0170
47	32	C28	CC	M	8.6530	2.2320	12.9390	-0.0490
48	47	C34	C*	B	8.3470	1.2730	13.8630	-0.2170
49	48	C31	C*	S	9.5030	1.0580	14.6600	-0.2170
50	49	H39	HA	E	9.5770	0.4460	15.3970	0.1480
51	48	H46	HA	E	7.5010	0.8300	13.9550	0.1480
52	47	N4	NA	M	9.9650	2.5970	13.1470	-0.1430
53	52	H38	H	E	10.4400	3.2590	12.6410	0.2690
54	52	C25	CC	M	10.4980	1.8800	14.2090	-0.0490
55	54	C23	CT	M	11.8500	2.1570	14.8080	0.0170
56	55	C26	CT	3	11.7290	3.3280	15.8330	0.0110
57	56	C32	CT	3	11.1840	4.6430	15.2840	-0.0430
58	57	H40	HC	E	11.1090	5.3610	15.9170	0.0150
59	57	H41	HC	E	10.3110	4.4680	14.9260	0.0150
60	57	H42	HC	E	11.7630	4.9300	14.5730	0.0150
61	56	H30	HC	E	11.1500	3.0410	16.5440	0.0170
62	56	H31	HC	E	12.6020	3.5030	16.1910	0.0170
63	55	C27	CT	3	12.3130	0.9310	15.6530	0.0110
64	63	C33	CT	3	12.5660	-0.3670	14.9020	-0.0430
65	64	H43	HC	E	12.8580	-1.1170	15.4260	0.0150
66	64	H44	HC	E	13.2340	-0.1940	14.2340	0.0150
67	64	H45	HC	E	11.7460	-0.6130	14.4670	0.0150
68	63	H32	HC	E	11.6450	0.7580	16.3200	0.0170
69	63	H33	HC	E	13.1320	1.1770	16.0870	0.0170
70	55	C19	CC	M	12.9050	2.4990	13.7830	-0.0490
71	70	C20	C*	B	13.9680	3.3520	13.8710	-0.2170
72	71	C14	C*	S	14.7270	3.2370	12.6820	-0.2170
73	72	H16	HA	E	15.5320	3.7190	12.4790	0.1480
74	71	H28	HA	E	14.1610	3.9330	14.6100	0.1480
75	70	N2	NA	M	13.0020	1.8710	12.5550	-0.1430
76	75	H15	H	E	12.3810	1.2170	12.2250	0.2690
77	75	C8	CC	M	14.1140	2.3160	11.8760	-0.0490
78	77	C4	CT	M	14.5580	1.7400	10.5580	0.0170
79	78	C9	CT	3	15.7260	0.7380	10.8040	0.0110
80	79	C15	CT	3	15.4930	-0.2790	11.9140	-0.0430
81	80	H17	HC	E	16.2160	-0.8960	12.0550	0.0150
82	80	H18	HC	E	15.3500	0.2150	12.7250	0.0150
83	80	H19	HC	E	14.6980	-0.7790	11.7110	0.0150
84	79	H6	HC	E	15.8690	0.2440	9.9930	0.0170
85	79	H7	HC	E	16.5110	1.2380	11.0080	0.0170
86	78	C7	CT	M	15.0740	2.9020	9.6570	0.0110
87	86	H4	HC	E	15.8280	3.3170	10.0840	0.0170
88	86	H5	HC	E	14.3640	3.5450	9.5820	0.0170
89	86	C13	CT	M	15.4870	2.5000	8.2480	-0.0430
90	89	H12	HC	E	15.7900	3.2230	7.6930	0.0150
91	89	H13	HC	E	14.7320	2.0860	7.8210	0.0150
92	89	H14	HC	E	16.1970	1.8580	8.3230	0.0150

NOYNAQg

1	0	O1	OH	M	11.4710	4.4260	11.1530	-0.6930
2	1	H2	HO	E	12.1680	3.8350	11.4680	0.4270
3	1	C1	CT	M	11.9960	5.7050	11.2150	0.1750
4	3	H3	H1	E	12.8240	5.6680	11.6980	0.0460
5	3	H4	H1	E	12.1700	6.0000	10.3170	0.0460
6	3	C2	CT	M	11.1450	6.6980	11.8530	0.1750
7	6	H5	H1	E	11.0180	6.4210	12.7630	0.0460
8	6	H6	H1	E	10.2990	6.6830	11.4010	0.0460
9	6	O2	OH	M	11.5650	7.9620	11.8870	-0.6930
10	9	H1	HO	E	10.8840	8.5100	12.2880	0.4270

OCAMIOh

1	0	C16	CT	M	9.6720	0.2030	30.5280	-0.2260
2	1	H9	HC	E	9.5170	-0.4310	29.7940	0.0610
3	1	H10	HC	E	9.3230	-0.2130	31.3200	0.0610
4	1	H11	HC	E	9.1360	0.9710	30.3350	0.0610
5	1	C13	CT	M	11.1800	0.5300	30.6410	0.1960

6	5	C17	CT	3	11.3600	1.4760	31.8320	-0.2260
7	6	H12	HC	E	10.8840	2.2970	31.7230	0.0610
8	6	H13	HC	E	11.0720	1.0770	32.6600	0.0610
9	6	H14	HC	E	12.2990	1.7030	31.9480	0.0610
10	5	C10	CA	M	11.9760	-0.7510	30.9180	0.0280
11	10	C7	CA	B	11.6160	-1.6150	31.9250	-0.1760
12	11	C4	CA	S	12.3590	-2.7410	32.2180	-0.2030
13	12	H2	HA	E	12.0610	-3.3600	32.9460	0.1660
14	11	H4	HA	E	10.8030	-1.4220	32.4310	0.1470
15	10	C6	CA	M	13.1170	-1.0890	30.2190	-0.1760
16	15	H3	HA	E	13.4070	-0.4820	29.5010	0.1470
17	15	C3	CA	M	13.8660	-2.2080	30.4960	-0.2030
18	17	H1	HA	E	14.6370	-2.4510	29.9290	0.1660
19	17	C2	CA	M	13.4740	-3.0200	31.5040	0.2850
20	19	O1	OS	M	14.1080	-4.2350	31.8680	-0.4640
21	20	C1	C	M	15.2850	-4.5970	31.3590	1.0630
22	21	O3	O	E	16.0250	-3.9400	30.7470	-0.6070
23	21	O2	OS	M	15.4740	-5.8730	31.6610	-0.4640
24	23	C5	CA	M	16.6260	-6.5010	31.1010	0.2850
25	24	C9	CA	B	16.4800	-7.2710	29.9900	-0.2030
26	25	C12	CA	S	17.5800	-7.8910	29.4620	-0.1760
27	26	H8	HA	E	17.4780	-8.4590	28.6480	0.1470
28	25	H6	HA	E	15.5890	-7.3610	29.5810	0.1660
29	24	C8	CA	M	17.8350	-6.3420	31.7100	-0.2030
30	29	H5	HA	E	17.9310	-5.8090	32.5370	0.1660
31	29	C11	CA	M	18.9400	-6.9910	31.1720	-0.1760
32	31	H7	HA	E	19.8110	-6.8990	31.6000	0.1470
33	31	C14	CA	M	18.8420	-7.7580	30.0160	0.0280
34	33	C18	CT	M	20.0430	-8.4010	29.3270	0.1960
35	34	C21	CT	3	19.7870	-9.9060	29.1020	-0.2260
36	35	H17	HC	E	20.4870	-10.3470	28.5990	0.0610
37	35	H18	HC	E	19.6630	-10.4040	29.9130	0.0610
38	35	H19	HC	E	18.9610	-10.0590	28.5740	0.0610
39	34	C22	CT	3	21.3290	-8.2950	30.1550	-0.2260
40	39	H20	HC	E	21.5490	-7.3870	30.3440	0.0610
41	39	H21	HC	E	21.2590	-8.7680	30.9820	0.0610
42	39	H22	HC	E	22.0850	-8.6780	29.6720	0.0610
43	34	C23	CA	M	20.2220	-7.6630	28.0040	0.0280
44	43	C27	CA	B	20.6510	-6.3180	28.0040	-0.1760
45	44	C30	CA	S	20.8000	-5.5950	26.8510	-0.2030
46	45	H28	HA	E	21.1370	-4.6630	26.8730	0.1660
47	44	H26	HA	E	20.8640	-5.9070	28.8730	0.1470
48	43	C26	CA	M	19.9440	-8.2130	26.7610	-0.1760
49	48	H25	HA	E	19.6830	-9.1550	26.7450	0.1470
50	48	C29	CA	M	20.0620	-7.4880	25.5890	-0.2030
51	50	H27	HA	E	19.8300	-7.8980	24.7230	0.1660
52	50	C31	CA	M	20.4860	-6.1920	25.6560	0.2850
53	52	O5	OS	M	20.6530	-5.5300	24.4100	-0.4640
54	53	C33	C	M	20.2700	-4.2570	24.2560	1.0630
55	54	O9	O	E	19.6970	-3.5870	25.0250	-0.6070
56	54	O8	OS	M	20.6630	-3.9160	23.0320	-0.4640
57	56	C37	CA	M	20.1360	-2.7210	22.4880	0.2850
58	57	C41	CA	B	18.8220	-2.6850	22.1080	-0.2030
59	58	C44	CA	S	18.3400	-1.5390	21.5190	-0.1760
60	59	H36	HA	E	17.3920	-1.5040	21.2260	0.1470
61	58	H34	HA	E	18.2150	-3.4190	22.3170	0.1660
62	57	C40	CA	M	20.9680	-1.6700	22.2850	-0.2030
63	62	H33	HA	E	21.9060	-1.7100	22.5680	0.1660
64	62	C43	CA	M	20.4570	-0.5170	21.6700	-0.1760
65	64	H35	HA	E	21.0470	0.2550	21.5060	0.1470
66	64	C46	CA	M	19.1390	-0.4350	21.2710	0.0280
67	66	C45	CT	M	18.5150	0.7840	20.5750	0.1960
68	67	C47	CT	3	18.0150	0.3420	19.2070	-0.2260
69	68	H37	HC	E	18.7000	-0.0170	18.6590	0.0610
70	68	H38	HC	E	17.3230	-0.3370	19.2940	0.0610
71	68	H39	HC	E	17.6050	1.0760	18.7240	0.0610
72	67	C48	CT	3	19.5530	1.9020	20.3470	-0.2260
73	72	H40	HC	E	19.1590	2.6800	19.9470	0.0610
74	72	H41	HC	E	19.9380	2.1800	21.1900	0.0610
75	72	H42	HC	E	20.2810	1.6200	19.7830	0.0610
76	67	C42	CA	M	17.4020	1.3130	21.4800	0.0280
77	76	C39	CA	B	17.7090	1.8500	22.7200	-0.1760
78	77	C36	CA	S	16.7410	2.3190	23.5790	-0.2030

79	78	H30	HA	E	16.9880	2.7130	24.4680	0.1660
80	77	H32	HA	E	18.6700	1.9130	22.9930	0.1470
81	76	C38	CA	M	16.0570	1.2470	21.1390	-0.1760
82	81	H31	HA	E	15.8150	0.8870	20.2600	0.1470
83	81	C35	CA	M	15.0650	1.6960	22.0050	-0.2030
84	83	H29	HA	E	14.1250	1.6090	21.7830	0.1660
85	83	C34	CA	M	15.4300	2.2150	23.2060	0.2850
86	85	O6	OS	M	14.3950	2.7410	24.0270	-0.4640
87	86	C32	C	M	14.2070	2.1720	25.2090	1.0630
88	87	O7	O	E	14.8300	1.3010	25.6880	-0.6070
89	87	O4	OS	M	13.1380	2.7570	25.7560	-0.4640
90	89	C28	CA	M	12.6930	2.2220	26.9800	0.2850
91	90	C24	CA	M	13.0030	2.7990	28.1550	-0.2030
92	91	H23	HA	E	13.6150	3.5830	28.1610	0.1660
93	91	C19	CA	M	12.4820	2.2860	29.3240	-0.1760
94	93	H15	HA	E	12.7100	2.7270	30.1830	0.1470
95	93	C15	CA	M	11.6600	1.1690	29.3430	0.0280
96	95	C20	CA	M	11.3440	0.6250	28.1070	-0.1760
97	96	H16	HA	E	10.7350	-0.1230	28.0810	0.1470
98	96	C25	CA	M	11.8590	1.1330	26.9220	-0.2030
99	98	H24	HA	E	11.5910	0.7330	26.0520	0.1660

OCAMIOg

1	0	O2	O	M	15.4200	-3.4110	27.0890	-0.5920
2	1	C1	C	M	16.4340	-3.5100	26.7610	0.8460
3	2	C3	CT	3	17.2830	-4.4910	27.6370	-0.3630
4	3	H3	HC	E	17.1640	-4.6160	28.6060	0.1030
5	3	H4	HC	E	18.2760	-4.3010	27.5690	0.1030
6	3	H5	HC	E	17.2070	-5.3800	27.2570	0.1030
7	2	O1	OS	M	16.9310	-2.4230	26.8770	-0.4890
8	7	C2	CT	M	16.1520	-1.5080	25.9530	0.2190
9	8	H1	H1	E	15.2260	-1.8100	25.8370	0.0280
10	8	H2	H1	E	16.1170	-0.6080	26.2390	0.0280
11	8	C4	CT	M	16.4530	-1.4260	24.7260	-0.0630
12	11	H6	HC	E	15.9710	-0.7760	24.1620	0.0260
13	11	H7	HC	E	17.4220	-1.0510	24.6320	0.0260
14	11	H8	HC	E	16.5030	-2.2300	24.2230	0.0260

UBETAWh

1	0	O2	O	M	11.5110	5.0420	8.3090	-0.5570
2	1	C2	C	M	11.3270	6.0820	7.7600	0.8170
3	2	C5	CT	3	10.0310	6.5530	7.1910	-0.3380
4	3	H1	HC	E	9.7560	7.3760	7.6220	0.1000
5	3	H2	HC	E	10.1310	6.7030	6.2390	0.1000
6	3	H3	HC	E	9.3590	5.8690	7.3420	0.1000
7	2	O1	OS	M	12.3130	7.0220	7.5980	-0.4040
8	7	C1	CA	M	13.5750	6.7300	8.1770	0.0960
9	8	C3	CA	M	13.7110	6.6310	9.5540	-0.0120
10	9	C6	CT	M	12.7480	6.8900	10.7130	0.0270
11	10	H4	HC	E	11.8120	6.7930	10.5230	0.0950
12	10	C11	CT	B	13.3510	5.9300	11.7570	-0.2510
13	12	H6	HC	E	12.9800	6.0580	12.6340	0.0920
14	12	H7	HC	E	13.2830	5.0080	11.4980	0.0920
15	10	C10	CA	M	13.2060	8.2200	11.2880	0.0080
16	15	C18	CA	B	12.6750	9.4610	11.2750	-0.2090
17	16	C22	CA	S	13.3950	10.5350	11.8580	0.0850
18	17	C26	CA	B	14.6860	10.3050	12.4190	0.0850
19	18	C21	CA	S	15.2210	8.9820	12.4080	-0.2090
20	19	H14	HA	E	16.0840	8.8030	12.7890	0.1400
21	18	C31	CA	B	15.3940	11.3910	12.9690	-0.1950
22	21	H21	HA	E	16.2570	11.2270	13.3590	0.1420
23	21	C35	CA	B	14.8670	12.6500	12.9780	-0.1380
24	23	C32	CA	B	13.6130	12.8870	12.4370	-0.1380
25	24	C27	CA	S	12.8820	11.8560	11.8750	-0.1950
26	25	H16	HA	E	12.0260	12.0330	11.4790	0.1420
27	24	H22	HA	E	13.2370	13.7690	12.4630	0.1360
28	23	H25	HA	E	15.3720	13.3760	13.3550	0.1360
29	16	H12	HA	E	11.8160	9.6130	10.8760	0.1400
30	15	C17	CA	M	14.5010	7.9740	11.8600	0.0080
31	30	C12	CT	M	14.7900	6.4920	11.6300	0.0270
32	31	H8	HC	E	15.4760	6.0900	12.1690	0.0950

33	31	C7	CA	M	14.9630	6.3740	10.1170	-0.0120
34	33	C13	CA	M	16.0860	6.2000	9.3150	0.0960
35	34	O3	OS	S	17.2480	5.6960	9.9100	-0.4040
36	35	C23	C	B	18.3310	6.4540	10.0600	0.8170
37	36	O4	O	E	18.3620	7.5950	9.7510	-0.5570
38	36	C28	CT	3	19.4570	5.6460	10.6370	-0.3380
39	38	H17	HC	E	20.2900	5.9150	10.2160	0.1000
40	38	H18	HC	E	19.5140	5.7960	11.5930	0.1000
41	38	H19	HC	E	19.2960	4.7050	10.4660	0.1000
42	34	C8	CA	M	15.9440	6.3530	7.9490	-0.0120
43	42	C4	CA	M	14.6900	6.6080	7.3900	-0.0120
44	43	C9	CT	M	14.9100	6.7680	5.8840	0.0270
45	44	H5	HC	E	14.1360	6.6750	5.3250	0.0950
46	44	C15	CT	M	16.0660	5.7470	5.6910	-0.2510
47	46	H10	HC	E	15.7930	4.8460	5.8750	0.0920
48	46	H11	HC	E	16.4750	5.7960	4.8230	0.0920
49	46	C14	CT	M	16.9580	6.3450	6.8110	0.0270
50	49	H9	HC	E	17.8000	5.9180	6.9780	0.0950
51	49	C19	CA	M	16.9960	7.7990	6.3180	0.0080
52	51	C24	CA	S	17.9650	8.7500	6.3660	-0.2090
53	52	H15	HA	E	18.8320	8.5420	6.7190	0.1400
54	51	C16	CA	M	15.7200	8.0700	5.7610	0.0080
55	54	C20	CA	M	15.4130	9.2890	5.2610	-0.2090
56	55	H13	HA	E	14.5430	9.4550	4.8860	0.1400
57	55	C25	CA	M	16.3860	10.3220	5.2960	0.0850
58	57	C29	CA	M	17.6820	10.0530	5.8510	0.0850
59	58	C33	CA	M	18.6250	11.0940	5.8950	-0.1950
60	59	H23	HA	E	19.4920	10.9150	6.2650	0.1420
61	59	C36	CA	M	18.3250	12.3480	5.4280	-0.1380
62	61	H26	HA	E	18.9750	13.0510	5.5050	0.1360
63	61	C34	CA	M	17.0740	12.6050	4.8600	-0.1380
64	63	H24	HA	E	16.8820	13.4680	4.4870	0.1360
65	63	C30	CA	M	16.1310	11.6270	4.8010	-0.1950
66	65	H20	HA	E	15.2700	11.8270	4.4260	0.1420

UBETAWg

1	0	C4	CA	M	15.0340	10.1300	8.6750	-0.1200
2	1	H5	HA	E	14.4460	9.3710	8.6590	0.1690
3	1	C1	CA	M	16.3550	9.9790	9.0120	0.0030
4	3	H1	H4	E	16.6910	9.1080	9.2380	0.2020
5	3	N1	N*	M	17.1920	11.0310	9.0230	0.0200
6	5	C3	CT	3	18.6270	10.8270	9.3830	-0.0400
7	6	H3	H1	E	19.1480	11.5680	9.0650	0.1070
8	6	H4	H1	E	18.9580	10.0230	8.9770	0.1070
9	6	C6	CT	3	18.7410	10.7410	10.8780	-0.0560
10	9	H7	HC	E	18.3110	11.5130	11.2790	0.0560
11	9	H8	HC	E	19.6730	10.7150	11.1430	0.0560
12	9	H9	HC	E	18.2970	9.9310	11.1780	0.0560
13	5	C2	CA	M	16.7460	12.2540	8.7230	0.0030
14	13	H2	H4	E	17.3520	12.9990	8.7360	0.2020
15	13	C5	CA	M	15.4370	12.4500	8.4050	-0.1200
16	15	H6	HA	E	15.1220	13.3330	8.1900	0.1690
17	15	C7	CA	M	14.5600	11.3830	8.3630	0.0280
18	17	C8	C	M	13.1530	11.6730	7.9580	0.7460
19	18	O1	O	E	12.7420	12.7720	7.7670	-0.5000
20	18	O2	OS	M	12.4590	10.5720	7.8390	-0.4260
21	20	C9	CT	M	11.0730	10.7430	7.3620	0.0920
22	21	H10	H1	E	10.8650	10.0020	6.7870	0.0880
23	21	H11	H1	E	11.0160	11.5490	6.8460	0.0880
24	21	C10	CT	M	10.2240	11.0710	8.4140	-0.0620
25	24	H12	HC	E	9.3000	11.0690	8.1200	0.0440
26	24	H13	HC	E	10.4700	11.9760	8.6830	0.0440
27	24	H14	HC	E	10.3300	10.4740	9.1680	0.0440

VOHVIXh

1	0	O1	OS	M	12.5540	18.4320	2.3550	-0.3170
2	1	C1	CT	M	11.4460	18.6900	3.2040	0.1680
3	2	H1	H1	E	11.1840	17.7730	3.7320	0.0180
4	2	H2	H1	E	11.7140	19.4680	3.9190	0.0180
5	2	C3	CT	M	10.2930	19.1320	2.4170	0.0470
6	5	H4	H1	E	9.5210	19.5030	3.0910	0.0410

7	5	H5	H1	E	10.6020	19.9270	1.7380	0.0410
8	5	O2	OS	M	9.7780	18.0550	1.6720	-0.2800
9	8	C8	CT	M	8.6320	18.4410	0.8830	0.0470
10	9	H13	H1	E	8.9010	19.3270	0.3080	0.0410
11	9	H14	H1	E	7.8220	18.6120	1.5920	0.0410
12	9	C11	CT	M	8.1630	17.4610	-0.0170	0.1680
13	12	H19	H1	E	7.3200	17.9200	-0.5330	0.0180
14	12	H20	H1	E	7.9060	16.5740	0.5610	0.0180
15	12	O4	OS	M	9.0550	17.0690	-1.0310	-0.3170
16	15	C13	CT	M	9.4430	17.9620	-2.0690	0.0150
17	16	H23	H1	E	9.9890	18.8000	-1.6360	0.0770
18	16	C15	CT	M	8.2620	18.5750	-2.8050	-0.0660
19	18	H26	HC	E	7.6470	19.1190	-2.0890	0.0340
20	18	H27	HC	E	8.6310	19.2520	-3.5750	0.0340
21	18	C18	CT	M	7.4120	17.4890	-3.4570	0.0320
22	21	H31	HC	E	7.0090	16.8320	-2.6860	-0.0010
23	21	H32	HC	E	6.5930	17.9530	-4.0060	-0.0010
24	21	C20	CT	M	8.2610	16.6830	-4.4120	0.0320
25	24	H35	HC	E	8.6170	17.3520	-5.1950	-0.0010
26	24	H36	HC	E	7.6560	15.8790	-4.8300	-0.0010
27	24	C19	CT	M	9.4710	16.0800	-3.7210	-0.0660
28	27	H33	HC	E	10.1010	15.5980	-4.4680	0.0340
29	27	H34	HC	E	9.1380	15.3480	-2.9850	0.0340
30	27	C16	CT	M	10.2750	17.1620	-3.0230	0.0150
31	30	H28	H1	E	10.6200	17.8210	-3.8200	0.0770
32	30	O6	OS	M	11.4060	16.6540	-2.3550	-0.3170
33	32	C17	CT	M	12.5150	16.3960	-3.2040	0.1680
34	33	H29	H1	E	12.2480	15.6180	-3.9190	0.0180
35	33	H30	H1	E	12.7780	17.3130	-3.7310	0.0180
36	33	C14	CT	M	13.6670	15.9540	-2.4170	0.0470
37	36	H24	H1	E	14.4390	15.5760	-3.0870	0.0410
38	36	H25	H1	E	13.3520	15.1650	-1.7340	0.0410
39	36	O5	OS	M	14.1820	17.0310	-1.6720	-0.2800
40	39	C12	CT	M	15.3290	16.6450	-0.8830	0.0470
41	40	H21	H1	E	16.1410	16.3980	-1.5670	0.0410
42	40	H22	H1	E	15.0040	15.8040	-0.2710	0.0410
43	40	C10	CT	M	15.7980	17.6250	0.0170	0.1680
44	43	H17	H1	E	16.6410	17.1660	0.5330	0.0180
45	43	H18	H1	E	16.0550	18.5120	-0.5610	0.0180
46	43	O3	OS	M	14.9060	18.0170	1.0310	-0.3170
47	46	C5	CT	M	14.5180	17.1240	2.0690	0.0150
48	47	H8	H1	E	13.9730	16.2860	1.6360	0.0770
49	47	C2	CT	M	13.6860	17.9240	3.0230	0.0150
50	49	H3	H1	E	13.3410	17.2650	3.8200	0.0770
51	49	C4	CT	M	14.4900	19.0060	3.7210	-0.0660
52	51	H6	HC	E	13.8610	19.5000	4.4620	0.0340
53	51	H7	HC	E	14.8300	19.7280	2.9790	0.0340
54	51	C6	CT	M	15.7000	18.4030	4.4120	0.0320
55	54	H9	HC	E	15.3590	17.7520	5.2170	-0.0010
56	54	H10	HC	E	16.3090	19.2190	4.8000	-0.0010
57	54	C9	CT	M	16.5490	17.5970	3.4570	0.0320
58	57	H15	HC	E	17.3680	17.1330	4.0060	-0.0010
59	57	H16	HC	E	16.9520	18.2540	2.6860	-0.0010
60	57	C7	CT	M	15.6980	16.5110	2.8050	-0.0660
61	60	H11	HC	E	15.3200	15.8480	3.5830	0.0340
62	60	H12	HC	E	16.3100	15.9520	2.0980	0.0340

VOHVIXg

1	0	N1	NT	M	11.5610	15.9850	0.4990	-0.9500
2	1	H1	H	E	11.0780	16.7550	1.1010	0.4320
3	1	H2	H	E	11.1240	15.9770	-0.5000	0.4320
4	1	S1	SY	M	11.3450	14.5590	1.1860	1.0770
5	4	O1	O	E	12.0040	13.5910	0.3450	-0.5600
6	4	O2	O	E	11.7470	14.7100	2.5500	-0.5600
7	4	C1	CA	M	9.6250	14.1850	1.1830	0.0170
8	7	C3	CA	B	8.8490	14.3310	2.3080	-0.1760
9	8	C5	CA	S	7.4880	14.0680	2.2750	-0.1600
10	9	H6	HA	E	6.8780	14.2070	3.1680	0.1450
11	8	H4	HA	E	9.3110	14.6580	3.2400	0.1850
12	7	C2	CA	M	9.0470	13.7330	0.0110	-0.1760
13	12	H3	HA	E	9.6510	13.5980	-0.8860	0.1850
14	12	C4	CA	M	7.6950	13.4550	-0.0070	-0.1600

15	14	H5	HA	E	7.2450	13.0880	-0.9300	0.1450
16	14	C6	CA	M	6.9060	13.6240	1.0880	0.1970
17	16	N2	N	M	5.5230	13.3260	0.9620	-0.4420
18	17	H7	H	E	5.1410	13.3140	-0.0590	0.2520
19	17	C7	C	M	4.6130	13.0550	1.9200	0.6590
20	19	C8	CT	3	3.2670	12.6560	1.4140	-0.3670
21	20	H8	HC	E	2.6060	12.4590	2.2580	0.1120
22	20	H9	HC	E	3.3590	11.7560	0.8070	0.1120
23	20	H10	HC	E	2.8530	13.4620	0.8080	0.1120
24	19	O3	O	M	4.8630	13.1100	3.1090	-0.5090

XIVVAZh

1	0	O6	OH	M	3.5380	-0.2220	7.6890	-0.5610
2	1	H20	HO	E	3.4710	0.7340	7.7470	0.4140
3	1	C13	CA	M	4.2140	-0.5680	6.5330	0.1630
4	3	C10	CA	M	4.0310	-1.8520	6.0150	-0.0240
5	4	C14	CA	S	4.8130	-2.2470	4.9300	-0.1620
6	5	H8	HA	E	4.7210	-3.1210	4.5700	0.1660
7	4	C8	CT	M	3.0100	-2.8290	6.5770	-0.0400
8	7	H5	HC	E	3.3210	-3.7540	6.4070	0.0520
9	7	H6	HC	E	2.9490	-2.7050	7.5560	0.0520
10	7	C5	CA	M	1.6250	-2.6500	5.9650	-0.0330
11	10	C7	CA	S	0.7130	-1.7370	6.4820	0.2740
12	11	O4	OH	S	1.0390	-1.0330	7.6310	-0.5880
13	12	H19	HO	E	1.2800	-1.6490	8.3270	0.4220
14	10	C3	CA	M	1.2350	-3.4440	4.8890	-0.1820
15	14	H2	HA	E	1.8420	-4.0730	4.5170	0.2110
16	14	C1	CA	M	-0.0430	-3.3120	4.3600	-0.1370
17	16	S1	SY	3	-0.6120	-4.5290	3.2040	1.1030
18	17	O1	O	E	-1.3660	-5.5040	4.0630	-0.5590
19	17	O2	O	E	0.5750	-5.1500	2.6700	-0.5590
20	17	O3	OH	S	-1.4840	-3.9200	2.2380	-0.5150
21	20	H22	HO	E	-1.7440	-4.5730	1.5840	0.4290
22	16	C2	CA	M	-0.9290	-2.3630	4.8550	-0.1820
23	22	H1	HA	E	-1.7870	-2.2660	4.4610	0.2110
24	22	C4	CA	M	-0.5620	-1.5500	5.9310	-0.0330
25	24	C6	CT	M	-1.5220	-0.5210	6.5000	-0.0400
26	25	H3	HC	E	-2.4520	-0.8120	6.3150	0.0520
27	25	H4	HC	E	-1.4080	-0.4810	7.4820	0.0520
28	25	C9	CA	M	-1.3150	0.8760	5.9180	-0.0240
29	28	C12	CA	S	-0.4250	1.8040	6.4820	0.1630
30	29	O5	OH	S	0.2260	1.4840	7.6560	-0.5610
31	30	H18	HO	E	0.4680	0.5550	7.6450	0.4140
32	28	C11	CA	M	-2.0510	1.2710	4.8230	-0.1620
33	32	H7	HA	E	-2.6580	0.6590	4.4220	0.1660
34	32	C15	CA	M	-1.9240	2.5550	4.2940	-0.1030
35	34	S2	SY	3	-2.8980	2.9960	2.8970	1.1930
36	35	O7	O	E	-2.9880	4.4460	2.8920	-0.6850
37	35	O8	O	E	-2.1620	2.4940	1.7370	-0.6850
38	35	O9	O	E	-4.1820	2.3320	3.0600	-0.6850
39	34	C19	CA	M	-1.0060	3.4510	4.8350	-0.1620
40	39	H9	HA	E	-0.9020	4.3140	4.4550	0.1660
41	39	C16	CA	M	-0.2380	3.0750	5.9400	-0.0240
42	41	C20	CT	M	0.7670	4.0730	6.5260	-0.0400
43	42	H10	HC	E	0.8010	3.9540	7.5080	0.0520
44	42	H11	HC	E	0.4490	4.9930	6.3440	0.0520
45	42	C23	CA	M	2.1690	3.9190	5.9650	-0.0330
46	45	C26	CA	B	2.5840	4.7140	4.9050	-0.1820
47	46	C28	CA	B	3.8760	4.5790	4.3910	-0.1370
48	47	C27	CA	S	4.7510	3.6230	4.8910	-0.1820
49	48	H16	HA	E	5.6160	3.5210	4.5100	0.2110
50	47	S4	SY	3	4.3450	5.6210	3.0340	1.1030
51	50	O14	OH	S	3.5650	5.1520	1.9010	-0.5150
52	51	H21	HO	E	3.3170	4.2350	2.0400	0.4290
53	50	O15	O	E	5.7770	5.4140	2.8420	-0.5590
54	50	O16	O	E	4.0090	6.9850	3.4180	-0.5590
55	46	H15	HA	E	1.9890	5.3520	4.5300	0.2110
56	45	C25	CA	M	3.0730	2.9930	6.4870	0.2740
57	56	O13	OH	S	2.7210	2.2750	7.6200	-0.5880
58	57	H17	HO	E	2.7840	2.8430	8.3920	0.4220
59	56	C24	CA	M	4.3520	2.8050	5.9680	-0.0330
60	59	C21	CT	M	5.3000	1.7500	6.5230	-0.0400

61	60	H12	HC	E	5.1840	1.7100	7.5070	0.0520
62	60	H13	HC	E	6.2300	2.0360	6.3440	0.0520
63	60	C17	CA	M	5.1080	0.3590	5.9600	-0.0240
64	63	C22	CA	M	5.8560	-0.0620	4.8720	-0.1620
65	64	H14	HA	E	6.4620	0.5420	4.4570	0.1660
66	64	C18	CA	M	5.7310	-1.3540	4.3760	-0.1030
67	66	S3	SY	M	6.7290	-1.8530	3.0150	1.1930
68	67	O11	O	E	8.0330	-1.2370	3.2010	-0.6850
69	67	O12	O	E	6.7640	-3.3140	3.0750	-0.6850
70	67	O10	O	M	6.0510	-1.3780	1.8110	-0.6850

XIVVAZg

1	0	O1	OH	M	2.5080	2.5470	0.5260	-0.5900
2	1	H11	HO	E	1.7860	3.1420	0.3120	0.5100
3	1	C1	C	M	2.4650	2.2660	1.7030	0.6290
4	3	O2	O	E	1.6050	2.7410	2.5650	-0.4480
5	3	C2	CT	M	3.4330	1.2640	2.3210	0.0060
6	5	N1	N3	3	4.3290	0.7780	1.2410	-0.4300
7	6	H2	H	E	4.8370	0.0910	1.5560	0.3600
8	6	H3	H	E	4.8710	1.4570	0.9670	0.3600
9	6	H4	H	E	3.8280	0.4880	0.5390	0.3600
10	5	H1	HP	E	3.9910	1.7490	2.9940	0.2070
11	5	C3	CT	M	2.6940	0.1530	3.0370	-0.2710
12	11	H5	HC	E	3.3510	-0.5460	3.2810	0.1760
13	11	H6	HC	E	2.3260	0.5190	3.8790	0.1760
14	11	C4	CC	M	1.5760	-0.4970	2.2790	0.0790
15	14	N2	NA	M	1.7030	-1.6860	1.5770	-0.1800
16	15	H7	H	E	2.4320	-2.1780	1.5290	0.3710
17	15	C6	CR	M	0.5500	-1.9620	0.9920	0.0330
18	17	H9	H5	E	0.3780	-2.7190	0.4440	0.2770
19	17	N3	NA	M	-0.3290	-1.0220	1.2910	-0.1540
20	19	H10	H	E	-1.1680	-0.9940	1.0270	0.3910
21	19	C5	CW	M	0.3040	-0.0890	2.0930	-0.0990
22	21	H8	H4	E	-0.0880	0.7000	2.4500	0.2380

YACVEEh

1	0	O2	OH	M	7.4000	1.4790	11.5510	-0.4880
2	1	H4	HO	E	7.2360	1.3510	10.7050	0.3610
3	1	C5	CA	M	6.5450	2.4630	11.9550	0.2940
4	3	C2	CA	M	5.7700	3.2120	11.0900	-0.0110
5	4	C6	CA	S	4.9060	4.1750	11.5690	-0.1320
6	5	H2	HA	E	4.3890	4.6810	10.9550	0.1390
7	4	S1	S	M	5.8360	2.9010	9.3370	-0.1780
8	7	C1	CA	M	7.4930	3.4170	8.9350	-0.0110
9	8	C4	CA	B	7.8360	4.7580	8.8860	-0.1320
10	9	C8	CA	B	9.0940	5.1940	8.4770	-0.1330
11	10	C11	CA	S	10.0050	4.2210	8.1020	-0.1320
12	11	H5	HA	E	10.8680	4.4820	7.8030	0.1390
13	10	C12	CT	3	9.3880	6.6980	8.3910	0.4130
14	13	C16	CT	3	10.8640	6.9700	8.0750	-0.2620
15	14	H7	HC	E	11.0780	6.5490	7.2510	0.0580
16	14	H8	HC	E	11.0230	7.9030	8.0040	0.0580
17	14	H9	HC	E	11.4090	6.6090	8.7650	0.0580
18	13	C17	CT	3	8.5430	7.2730	7.2670	-0.2620
19	18	H10	HC	E	8.7020	8.2060	7.1920	0.0580
20	18	H11	HC	E	7.6260	7.1220	7.4620	0.0580
21	18	H12	HC	E	8.7680	6.8460	6.4500	0.0580
22	13	C18	CT	3	9.0640	7.3880	9.7150	-0.2620
23	22	H13	HC	E	9.2260	8.3210	9.6390	0.0580
24	22	H14	HC	E	8.1500	7.2410	9.9240	0.0580
25	22	H15	HC	E	9.6100	7.0280	10.4050	0.0580
26	9	H1	HA	E	7.1880	5.4040	9.1390	0.1390
27	8	C3	CA	M	8.4300	2.4570	8.5940	0.2940
28	27	O1	OH	S	8.0790	1.1470	8.7010	-0.4880
29	28	H3	HO	E	8.7010	0.7160	8.6770	0.3610
30	27	C7	CA	M	9.6900	2.8560	8.1490	-0.0110
31	30	S2	S	M	10.7910	1.6200	7.5200	-0.1780
32	31	C15	CA	M	12.0290	1.4370	8.7810	-0.0110
33	32	C24	CA	S	11.7370	1.0190	10.0760	0.2940
34	33	O3	OH	S	10.4280	0.8130	10.4270	-0.4880
35	34	H27	HO	E	10.2140	1.1140	11.1920	0.3610

36	32	C23	CA	M	13.3500	1.6340	8.3940	-0.1320
37	36	H25	HA	E	13.5310	1.9170	7.5060	0.1390
38	36	C27	CA	M	14.4120	1.4230	9.2730	-0.1330
39	38	C32	CT	3	15.8520	1.6310	8.7930	0.4130
40	39	C35	CT	3	16.0570	3.1050	8.4480	-0.2620
41	40	H31	HC	E	16.9410	3.2360	8.1220	0.0580
42	40	H32	HC	E	15.4320	3.3670	7.7830	0.0580
43	40	H33	HC	E	15.9270	3.6320	9.2290	0.0580
44	39	C36	CT	3	16.8700	1.2390	9.8360	-0.2620
45	44	H34	HC	E	16.7380	1.7690	10.6140	0.0580
46	44	H35	HC	E	16.7540	0.3220	10.0540	0.0580
47	44	H36	HC	E	17.7480	1.3780	9.5020	0.0580
48	39	C37	CT	3	16.1000	0.7960	7.5310	-0.2620
49	48	H37	HC	E	15.4760	1.0440	6.8580	0.0580
50	48	H38	HC	E	16.9820	0.9480	7.2140	0.0580
51	48	H39	HC	E	15.9920	-0.1240	7.7400	0.0580
52	38	C31	CA	M	14.1050	1.0080	10.5560	-0.1320
53	52	H29	HA	E	14.8090	0.8520	11.1720	0.1390
54	52	C28	CA	M	12.7840	0.7960	10.9610	-0.0110
55	54	S4	S	M	12.3960	0.1500	12.5860	-0.1780
56	55	C38	CA	M	13.7370	0.7800	13.5770	-0.0110
57	56	C44	CA	B	14.8910	0.0350	13.7540	-0.1320
58	57	C48	CA	B	15.9700	0.5070	14.5030	-0.1330
59	58	C51	CA	S	15.7920	1.7280	15.1580	-0.1320
60	59	H53	HA	E	16.4840	2.0440	15.7270	0.1390
61	58	C52	CT	3	17.3080	-0.2390	14.5260	0.4130
62	61	C55	CT	3	17.1020	-1.7370	14.7290	-0.2620
63	62	H55	HC	E	16.6950	-1.8880	15.5730	0.0580
64	62	H56	HC	E	17.9410	-2.1810	14.6980	0.0580
65	62	H57	HC	E	16.5400	-2.0750	14.0420	0.0580
66	61	C56	CT	3	18.0140	-0.0120	13.1920	-0.2620
67	66	H58	HC	E	17.4680	-0.3350	12.4840	0.0580
68	66	H59	HC	E	18.1640	0.9200	13.0770	0.0580
69	66	H60	HC	E	18.8450	-0.4700	13.1850	0.0580
70	61	C57	CT	3	18.2160	0.2760	15.6300	-0.2620
71	70	H61	HC	E	17.7990	0.1310	16.4710	0.0580
72	70	H62	HC	E	19.0460	-0.1820	15.6050	0.0580
73	70	H63	HC	E	18.3630	1.2070	15.5060	0.0580
74	57	H49	HA	E	14.9490	-0.8220	13.3470	0.1390
75	56	C43	CA	M	13.6100	2.0490	14.1790	0.2940
76	75	O5	OH	S	12.5430	2.8680	14.0070	-0.4880
77	76	H51	HO	E	11.9220	2.5160	13.5030	0.3610
78	75	C47	CA	M	14.6520	2.4960	15.0120	-0.0110
79	78	S6	S	M	14.6050	4.1130	15.7360	-0.1780
80	79	C49	CA	M	13.3070	4.0530	16.9390	-0.0110
81	80	C53	CA	B	13.5390	3.5450	18.2270	-0.1320
82	81	C50	CA	B	12.6180	3.6710	19.2510	-0.1330
83	82	C46	CA	S	11.4350	4.3330	18.9570	-0.1320
84	83	H50	HA	E	10.7920	4.4390	19.6480	0.1390
85	82	C54	CT	3	12.8980	3.2050	20.6820	0.4130
86	85	C58	CT	3	12.7740	4.4030	21.6140	-0.2620
87	86	H64	HC	E	13.4250	5.0570	21.3830	0.0580
88	86	H65	HC	E	11.9050	4.7750	21.5210	0.0580
89	86	H66	HC	E	12.9080	4.1300	22.5150	0.0580
90	85	C59	CT	3	14.2400	2.5640	20.8230	-0.2620
91	90	H67	HC	E	14.2890	1.8100	20.2480	0.0580
92	90	H68	HC	E	14.9030	3.1960	20.5730	0.0580
93	90	H69	HC	E	14.3900	2.2880	21.7210	0.0580
94	85	C60	CT	3	11.8770	2.1560	21.0780	-0.2620
95	94	H70	HC	E	12.0320	1.8770	21.9720	0.0580
96	94	H71	HC	E	11.0130	2.5450	21.0120	0.0580
97	94	H72	HC	E	11.9320	1.4050	20.4980	0.0580
98	81	H54	HA	E	14.3540	3.0910	18.4010	0.1390
99	80	C45	CA	M	12.0980	4.6880	16.6670	0.2940
100	99	O6	OH	S	11.7900	5.1950	15.4470	-0.4880
101	100	H52	HO	E	12.2970	4.8770	14.9330	0.3610
102	99	C39	CA	M	11.1470	4.8330	17.7030	-0.0110
103	102	S5	S	M	9.6550	5.7500	17.4080	-0.1780
104	103	C29	CA	M	8.5040	4.4110	17.2190	-0.0110
105	104	C33	CA	B	7.6730	4.0420	18.2800	-0.1320
106	105	C30	CA	B	6.7600	3.0070	18.1760	-0.1330
107	106	C26	CA	S	6.7250	2.3160	16.9550	-0.1320
108	107	H26	HA	E	6.1260	1.5880	16.8570	0.1390

109	106	C34	CT	3	5.8180	2.6200	19.3160	0.4130
110	109	C40	CT	3	4.3620	2.8860	18.8710	-0.2620
111	110	H40	HC	E	4.1780	2.3830	18.0870	0.0580
112	110	H41	HC	E	3.7660	2.6240	19.5630	0.0580
113	110	H42	HC	E	4.2470	3.8110	18.6890	0.0580
114	109	C41	CT	3	5.9830	1.1530	19.6410	-0.2620
115	114	H43	HC	E	6.8780	0.9850	19.9150	0.0580
116	114	H44	HC	E	5.7860	0.6400	18.8660	0.0580
117	114	H45	HC	E	5.3880	0.9100	20.3410	0.0580
118	109	C42	CT	3	6.0490	3.4430	20.5750	-0.2620
119	118	H46	HC	E	5.4440	3.1740	21.2560	0.0580
120	118	H47	HC	E	5.9200	4.3640	20.3820	0.0580
121	118	H48	HC	E	6.9410	3.3050	20.8720	0.0580
122	105	H30	HA	E	7.7330	4.5230	19.0970	0.1390
123	104	C25	CA	M	8.4170	3.7240	16.0050	0.2940
124	123	O4	OH	S	9.1980	4.0490	14.9380	-0.4880
125	124	H28	HO	E	9.7230	4.6710	15.2320	0.3610
126	123	C19	CA	M	7.5210	2.6690	15.8800	-0.0110
127	126	S3	S	M	7.4840	1.7180	14.3790	-0.1780
128	127	C9	CA	M	6.4780	2.7360	13.3140	-0.0110
129	128	C13	CA	M	5.5980	3.6980	13.7910	-0.1320
130	129	H6	HA	E	5.5550	3.8660	14.7240	0.1390
131	129	C10	CA	M	4.7800	4.4350	12.9280	-0.1330
132	131	C14	CT	M	3.7490	5.4440	13.4130	0.4130
133	132	C21	CT	3	2.3540	4.8820	13.0420	-0.2620
134	133	H19	HC	E	1.6810	5.4830	13.3410	0.0580
135	133	H20	HC	E	2.2290	4.0360	13.4560	0.0580
136	133	H21	HC	E	2.2950	4.7850	12.0990	0.0580
137	132	C22	CT	3	3.8270	5.6950	14.9110	-0.2620
138	137	H22	HC	E	4.6800	6.0580	15.1220	0.0580
139	137	H23	HC	E	3.7070	4.8770	15.3800	0.0580
140	137	H24	HC	E	3.1500	6.3090	15.1680	0.0580
141	132	C20	CT	M	3.9280	6.7710	12.6840	-0.2620
142	141	H16	HC	E	4.7970	7.1090	12.8690	0.0580
143	141	H17	HC	E	3.8320	6.6410	11.7490	0.0580
144	141	H18	HC	E	3.2750	7.3930	12.9860	0.0580

YACVEEg

1	0	O1	OH	M	10.2360	2.6850	12.5030	-0.6550
2	1	H1	HO	E	9.7110	2.5660	13.0630	0.4210
3	1	C1	CT	M	9.9780	3.9070	11.7780	0.2360
4	3	H2	H1	E	9.2700	4.3770	12.2030	0.0310
5	3	H3	H1	E	9.7280	3.6950	10.8870	0.0310
6	3	C2	CA	M	11.2020	4.7950	11.7530	-0.0040
7	6	C3	CA	M	12.2900	4.4900	10.9360	-0.1490
8	7	H4	HA	E	12.2540	3.7310	10.3650	0.1300
9	7	C5	CA	M	13.4120	5.2890	10.9390	-0.1540
10	9	H6	HA	E	14.1440	5.0800	10.3680	0.1400
11	9	C7	CA	M	13.4820	6.3890	11.7640	-0.1240
12	11	H8	HA	E	14.2630	6.9280	11.7690	0.1310
13	11	C6	CA	M	12.4200	6.7060	12.5720	-0.1540
14	13	H7	HA	E	12.4660	7.4630	13.1440	0.1400
15	13	C4	CA	M	11.2950	5.9100	12.5640	-0.1490
16	15	H5	HA	E	10.5570	6.1400	13.1180	0.1300
