# Supplementary material for: Normal-Mode-Analysis–Monitored Energy Minimization Procedure for Generating Small–Molecule Bound Conformations
Source: PLoS One. 2007 Oct 10;2(10):e1025. doi: 10.1371/journal.pone.0001025 (PMC1995756; doi:10.1371/journal.pone.0001025)
Supplement: Table S2 — The AMBER force field parameters for the 22 guest structures. (0.02 MB PDF) [file pone.0001025.s003.pdf]

Table S2. The AMBER force field parameters for the 22 guest structures.

MASS		
CA	12.01	0.36
CC	12.01	0.36
CM	12.01	0.36
CR	12.01	0.36
CV	12.01	0.36
CW	12.01	0.36
C	12.01	0.616
CT	12.01	0.878
H1	1.008	0.135
H4	1.008	0.135
H5	1.008	0.135
HA	1.008	0.135
HC	1.008	0.135
HO	1.008	0.135
HP	1.008	0.135
H	1.008	0.161
NH	14.01	0.53
N	14.01	0.53
N*	14.01	0.53
N2	14.01	0.53
N3	14.01	0.53
NA	14.01	0.53
NB	14.01	0.53
NT	14.01	0.53
O	16	0.434
OH	16	0.465
OS	16	0.465
P	30.97	1.538
S	32.06	2.9
SS	32.06	2.9
SY	32.06	2.9
BOND		
CA-SY	247.7	1.784
CM-SS	281.5	1.734
CM-N2	320.6	1.47
C-SS	328.9	1.675
OS-P	342.5	1.602
C-CV	346.5	1.49
CM-C	346.5	1.49
NT-SY	353.8	1.632
OS-CA	364	1.38
H-NH	401.2	1.014
N2-OH	416.2	1.394
NB-S	433.5	1.56
CA-N*	440.2	1.37
CA-NH	449	1.364
CR-NH	449	1.364
N-N	469.7	1.39
CM-CM	473.7	1.39
CV-CR	473.7	1.39
CM-N	478.2	1.345
CM-NT	478.2	1.345
O-P	487.7	1.481
O-SY	493	1.466
C-O	540.4	1.26
OH-HO	553.2	0.96
N2-O	789.9	1.209
ANGLE		
C-CT-CA	64.161	110.965
C-CT-OH	66.939	111.33
C-CV-NB	64.993	120

CA-C-OS	67.662	110.765
CV-NB-S	74.15	118.065
N3-CT-CA	65.679	111.845
OH-CT-CA	67.122	110.725
S-NB-CR	74.15	118.065
C-CM-CM	62	122.13
C-CM-N2	68.4	120.89
C-CV-CR	62	122.13
C-N-H	49.2	118.46
CA-C-O	67.1	126.4
CA-CA-N*	68.7	111.2
CA-N*-CA	67.7	110.55
CA-N*-CT	62.7	123.21
CM-C-O	67.1	126.4
CM-CM-N	69.9	104.81
CM-CM-N2	66.8	119.57
CM-CM-SS	66.8	122.34
CM-N-H	49.2	118.46
CM-N-N	68.2	118.42
CM-N2-O	77.4	112.16
CM-N2-OH	72.6	109.87
CM-NT-H	49.2	118.46
CM-NT-N	68	117.06
H1-CT-CA	50	109.5
HO-OH-N2	50.6	102.74
HP-CT-CA	50	109.5
N-C-CA	68	112.68
N-C-CV	68	112.68
N-CM-N	75.4	111.7
N-CM-SS	65.7	123.88
NT-CM-CM	69.5	121.19
NT-CM-SS	65.7	123.88
O-C-CA	67.1	126.4
O-C-CV	67.1	126.4
OS-CA-CA	66.1	122.03
CA-SY-NT	67.3	107.49
CT-N-CA	62.4	121.15
NB-S-NB	74.4	99.5
C-N-C	67.4	119.63
C-N-CA	64.3	123.71
C-N-CM	65.2	124.19
C-N-N	68.2	118.42
CA-CA-NH	69.3	120.13
CA-CA-SY	61.6	119.89
CA-N-H	47.6	114.59
CA-NH-H	49.6	113.79
CA-OS-P	78.5	118
CA-SY-O	65.8	108.73
CM-C-N	70.2	111.86
CR-CV-NB	71.1	112.56
CR-NH-H	49.6	113.79
CV-CR-NB	71.1	112.56
CV-CR-NH	69.3	120.13
H-NH-H	40.1	114.43
H-NT-SY	45.4	112.52
N-C-N	75.4	111.7
N-C-SS	65.7	123.88
N-CM-N2	70.9	123.86
NB-CR-NH	72.4	120.11
NT-SY-O	72.9	106.8
O-P-O	46	115.8
O-P-OS	44	116.09
O-SY-O	72.4	121.88

DIHEDRAL

X-OS-CA-X	1	0.9	180	2

X-NB-S-X	1	1.3	180	1
X-CA-N-X	4	1.8	180	2
X-N2-OH-X	1	3.2	180	2
X-C-CM-X	1	3.625	180	2
X-C-CV-X	1	3.625	180	2
X-CV-CR-X	1	3.625	180	2
X-CA-NH-X	4	4.2	180	2
X-CR-NH-X	4	4.2	180	2
X-N-N-X	4	4.6	0	2
X-N-CM-X	4	6.6	180	2
X-CA-SY-X	6	7.8	180	2
X-CM-N2-X	2	8.3	180	2
X-CM-NT-X	4	10	180	2
X-NT-SY-X	6	18.8	0	2

IMPROPER

CA-H1-CA-N*	1.1	180	2
CA-H4-CA-N*	1.1	180	2
CM-N-CM-SS	1.1	180	2
CT-CW-CC-NA	1.1	180	2
NT-NT-CM-SS	1.1	180	2

NONBOND

NH	1.824	0.17
SY	2	0.25
