# Supplementary material for: Normal-Mode-Analysis–Monitored Energy Minimization Procedure for Generating Small–Molecule Bound Conformations
Source: PLoS One. 2007 Oct 10;2(10):e1025. doi: 10.1371/journal.pone.0001025 (PMC1995756; doi:10.1371/journal.pone.0001025)
Supplement: Table S3 — Detailed information with regard to the docking studies of the 22 host-guest complexes. (0.02 MB PDF) [file pone.0001025.s004.pdf]

Table S3. Detailed information with regard to the docking studies of the 22 host-guest complexes.

CSD code*	host atom #	guest atom #	R factor	cubic box size (Å)	energy cutoff (kcal/mol)
AJUXOS	62	10	4.21	6	-10
AJUXUY	42	14	3.34	6	-10
AJUYAF	62	14	3.03	6	-10
BAFZEN	52	14	5.20	6	-30
BAPRAM	42	24	5.37	6	-10
BAPREQ	42	19	4.66	6	-10
BEGVOZ	46	15	4.74	6	-45
CECMEC10	180	19	5.50	6	-20
DESHEO	43	18	6.50	6	-20
DOXWAO	74	24	8.60	6	-30
FANJAG	90	23	7.37	6	-10
GUGGUK	67	14	4.77	6	-160
HASWUT	59	16	5.26	6	-90
JEJWOK	180	19	7.70	6	-10
KAXPOO	69	29	5.60	6	-10
LAYMAZ	48	22	4.40	6	-20
NOYNAQ	92	10	7.17	6	-10
OCAMIO	99	14	6.80	6	-10
UBETAW	66	27	4.62	6	-20
VOHVIX	62	24	7.50	6	-20
XIVVAZ	70	22	6.83	6	-60
YACVEE	144	16	7.50	6	-20

* Cambridge Structural Database code
